# Ribosomal Protein RPL10A Contributes to Early Plant Development and Abscisic Acid-Dependent Responses in *Arabidopsis*

**DOI:** 10.3389/fpls.2020.582353

**Published:** 2020-11-05

**Authors:** Rocío Soledad Ramos, Paula Casati, Claudia Patricia Spampinato, María Lorena Falcone Ferreyra

**Affiliations:** Centro de Estudios Fotosintéticos y Bioquímicos (CEFOBI), Facultad de Ciencias Bioquímicas y Farmacéuticas, Universidad Nacional de Rosario, Rosario, Argentina

**Keywords:** seed germination, ABA, early seedling development, *rpl10A* mutant, *RPL10A*-overexpressing lines

## Abstract

Plant ribosomal proteins play universal roles in translation, although they are also involved in developmental processes and hormone signaling pathways. Among *Arabidopsis* RPL10 family members, *RPL10A* exhibits the highest expression during germination and early development, suggesting that RPL10A is the main contributor to these processes. In this work, we first analyzed *RPL10A* expression pattern in *Arabidopsis thaliana* using transgenic *RPL10A_pro_:GUS* plants. The gene exhibits a ubiquitous expression pattern throughout the plant, but it is most strongly expressed in undifferentiated tissues. Interestingly, gene expression was also detected in stomatal cells. We then examined protein function during seedling establishment and abscisic acid (ABA) response. Heterozygous *rpl10A* mutant plants show decreased ABA-sensitivity during seed germination, are impaired in early seedling and root development, and exhibit reduced ABA-inhibition of stomatal aperture under light conditions. Overexpression of *RPL10A* does not affect the germination and seedling growth, but *RPL10A*-overexpressing lines are more sensitive to ABA during early plant development and exhibit higher stomatal closure under light condition both with and without ABA treatment than wild type plants. Interestingly, *RPL10A* expression is induced by ABA. Together, we conclude that RPL10A could act as a positive regulator for ABA-dependent responses in *Arabidopsis* plants.

## Introduction

Plant ribosomal proteins exist as families of two or more members, which are incorporated into the ribosome in certain tissues or under particular situations (Byrne, 2009; Moin et al., 2016a). Several evidences indicate that, in eukaryotes, the ribosome heterogeneity conferred by different ribosomal components allows the selective translation of specific mRNAs (Byrne, 2009; Moin et al., 2016a; Calamita et al., 2018). The characterization of *Arabidopsis thaliana* ribosomal protein mutant plants, as well as the complementation observed between paralog genes, has demonstrated partial and even absence of redundancy between family members. Each family member acts specifically at different developmental stages, in different tissues or under particular stress conditions (Sormani et al., 2011; Horiguchi et al., 2012; Hummel et al., 2012). *Arabidopsis* mutants in genes encoding ribosomal proteins exhibit a wide range of developmental phenotypes, including extreme anomalies such as embryo lethality, suggesting that ribosomes could also have specific functions during development (Degenhardt and Bonham-Smith, 2008; Horiguchi et al., 2012; Carroll, 2013).

Under sub-optimal environmental conditions, such as hypoxia, salinity, and heat, different studies have shown that the abundance of a transcript does not necessarily correlate with its association with polysomes and, therefore, with its translational level (Juntawong et al., 2014; Yamasaki et al., 2015). Furthermore, the absence of correlation between transcription and translation was demonstrated in *Arabidopsis* seeds during imbibition and germination (Basbouss-Serhal et al., 2015). During germination, gene expression is mainly regulated at a translational level and involves the selective and dynamic recruitment of specific mRNA to polysomes (Basbouss-Serhal et al., 2015). In this way, components of the translational machinery, such as ribosomal proteins, are key players in the regulation of the germination process.

The ribosomal protein L10 (RPL10), a component of the major subunit (60S) of the ribosome, is a key factor to form the 80S functional ribosome (Hofer et al., 2007). *Arabidopsis* has three genes encoding RPL10 proteins, *RPL10A*, *RPL10B*, and *RPL10C*. Previously, we have demonstrated that *Arabidopsis RPL10* genes are differentially regulated by UV-B radiation and are not functionally equivalent (Falcone Ferreyra et al., 2010a). Homozygous *rpl10A* mutants are not viable, indicating that RPL10A is essential for plant survival; homozygous *rpl10B* plants exhibit abnormal growth and development in both their vegetative and reproductive stages, while homozygous *rpl10C* plants do not exhibit phenotypic differences compared to wild type plants under normal growth conditions. All the three RPL10 proteins are localized both in the cytosol and the nuclei, suggesting that these proteins have extra-ribosomal functions (Falcone Ferreyra et al., 2013). This observation is supported by other studies, which have also proposed RPL10 extra-ribosomal roles in several eukaryotic organisms, including plants, such as transcriptional regulation under stress responses (Singh et al., 2009; Zorzatto et al., 2015; Moin et al., 2016a,b; Shi et al., 2017).

Previous reports indicate that RPL10A plays a predominant role during abiotic and biotic stresses (Imai et al., 2008; Falcone Ferreyra et al., 2010a, 2013; Zorzatto et al., 2015), and published microarray and RNA-seq gene expression data show that *RPL10A*, among *Arabidopsis RPL10* family members, exhibits the highest expression during germination and early development (Klepikova et al., 2016). Moreover, plant ribosomal proteins are also involved in different hormone signaling pathways (Cherepneva et al., 2003; Guo et al., 2011; Rosado et al., 2012; Moin et al., 2016b; Denver and Ullah, 2019). Here, we aim to gain further insights in the contribution of RPL10A during plant development and abscisic acid (ABA) responses.

ABA plays important roles during plant growth and development, including seed dormancy and germination, vegetative growth, radicular architecture, and stomatal movement (Sah et al., 2016; Santos Teixeira and ten Tusscher, 2019). In addition, ABA promotes leaf senescence and regulates responses to both biotic and abiotic stresses. Low water availability resulting from drought, high salinity, and cold promotes ABA synthesis, which stimulates stomatal closure and modulates gene expression changes so that plants can cope with stressful conditions (Ng et al., 2014; Sah et al., 2016).

Our data show that *RPL10A* is ubiquitously expressed throughout *A. thaliana*, but it is most strongly expressed in undifferentiated tissues. Interestingly, gene expression was also detected in stomatal cells. We then examined the role of the protein in regulating germination and seedling development. We have used heterozygous *rpl10A* mutant and *RPL10A*-overexpressing lines. We demonstrate that the mutant and overexpressing lines are less and more sensitive to ABA, respectively, than wild type plants, evidenced by an altered germination percentage, cotyledon greening percentage, primary root elongation, lateral root (LR) density, petiole length, leaf number, chlorophyll content, and water loss. Even more, *RPL10A* transcript levels do not increase in *abi3* and *abi5* mutants as in wild type seedlings. These results suggest that RPL10A is required for some ABA-dependent responses.

## Materials and Methods

### Plant Material and Growth Conditions

*Arabidopsis thaliana* ecotypes, Columbia-0 (Col-0, hereinafter referred to as WT), Wassilewskja (hereinafter referred to as WT Ws), and Landsberg erecta (hereinafter referred to as WT Ler), were used in this study. Seeds were vernalized for 3 days at 4°C in plates containing MS medium (Murashige and Skoog plant salt mixture) supplemented with 0.7% (w/v) agar or in pots containing soil, as indicated. Plates and pots were then placed in a growth chamber under a light intensity of 100 μE m^−2^ s^−1^ with a photoperiod of 16-h-light/8-h-dark at 22°C. The *abi3-1* mutant (CS24, background Ler-0) and the *abi5-1* mutant (CS8105, background Ws) were obtained from the *Arabidopsis* Biological Resource Center (ABRC, Columbus, OH). The *rpl10A* heterozygous mutant lines (SALK 010170 and SALK 106656, designed *rpl10A-1* and *rpl10A-2*, respectively) and transgenic *RPL10A_pro_:GUS* plants were previously described (Falcone Ferreyra et al., 2013).

For ABA treatment in MS liquid medium, seedlings previously grown on MS-agar plates for 14 days were transferred to MS liquid medium supplemented or not with 5, 10, and 20 μM ABA and incubated under light conditions (with a photoperiod of 16-h-light/8-h-dark) with gentle agitation. Afterwards, seedlings were collected at 4 and 24 h after treatment and stored at −80°C until use.

### Germination and Early Plant Development Assays

*Arabidopsis thaliana* seeds were surface sterilized, sown on MS-agar plates with or without ABA supplementation, as indicated. After stratification at 4°C for 3 days in the dark, plates were transferred to a grow chamber at 22°C, under a photoperiod of 16-h-light/8-h-dark and a light intensity of 100 μE m^−2^ s^−1^. Germination was monitored at the indicated times (h) by counting the frequency of radicle emergence through the seed coat. The percentage of inhibition of germination by exogenous ABA was calculated using the following formula: [(Germination % in MS – Germination % in MS + ABA) / (Germination % in MS)] × 100. Cotyledon greening is defined as the full opening of green cotyledons, while seedling stage is recorded when leaf #4 is emerging. Experiments were performed in triplicate, with at least 100 seeds each. For all genotypes analyzed, seeds had same after-ripening time periods.

For analysis of the ABA effect after germination, seedlings previously grown on MS-agar plates for 7 days were transferred to MS-agar medium supplemented or not with 5 and 10 μM ABA. Leaf numbers, petiole lengths, and chlorophyll contents were measured over time for 10 days. Seedlings were photographed 8 and 10 days after the transfer.

### GUS Histochemical Staining in Transgenic *Arabidopsis* Plants

Three independent T3 transgenic lines (*RPL10A_pro_:GUS*) were used for histochemical analysis. Transgenic *RPL10A_pro_:GUS* seeds were sown in MS-agar plates. Samples were collected at different developmental stages (germinative and post-germinative) to further stain with 5-bromo-4-chloro-3-indolyl-d-glucuronide at 37°C for 24 h, followed by washes with ethanol. Seedlings were kept in 50% (v/v) ethanol and 5% (v/v) acetic acid before being photographed. Germinated seed stage is defined as a visible radicle protrusion through the seed coat. Samples were collected for staining at 22 or 46 h after stratification in the absence or presence of ABA, respectively. Cotyledon greening stage is defined as the full opening of green cotyledons. Samples were collected for staining at 4 or 8 days after stratification in the absence or presence of ABA, respectively.

For guard cells staining, 8-day-old seedlings were first stained and abaxial epidermis was further peeled from leaves as described below. The experiments were repeated three times using at least three independent lines with similar results.

### Plant Transformation

To generate *RPL10A*-overexpressing plants, the full-length ORF of *RPL10A* was amplified by PCR using F-*Kpn*I*-AtRPL10A* and R-*Sal*I-*AtRPL10A* primers containing the *Kpn*I and *Sal*I restriction sites for cloning and the start and stop codons, respectively (Supplementary Table S1; Falcone Ferreyra et al., 2013). PCR reaction was performed using Platinum Pfx DNA polymerase (Invitrogen) under the following conditions: 1X Pfx buffer, 1.5 mM MgSO_4_, 0.5 μM of each primer, 0.2 mM of each dNTP, 0.5 U Platinum Pfx DNA polymerase, and the plasmid pCHF_3_:*AtRPL10A-GFP* as template in 25 μl of final volume. Cycling conditions were as follows: 60 s denaturation at 95°C, followed by 35 cycles of 20 s denaturation at 95°C, 30 s annealing at 60°C, 60 s amplification at 68°C, and a final extension of 7 min at 68°C. The PCR product was purified, cut with the corresponding *Kpn*I and *Sal*I restriction enzymes, purified and cloned into pCS052_GFP_pCHF3 vector (a modified version of pCHF3; Falcone Ferreyra et al., 2013) previously cut with the same enzymes, generating *35S_pro_:RPL10A* construct. The absence of random mutations in the PCR amplified fragment was determined by DNA sequencing. The *35S_pro_:RPL10A* construct was transformed into *Agrobacterium tumefaciens* strain GV3101 by electroporation, and the transformation of *A. thaliana* plants (WT) by the resulting bacteria was performed by the floral dip method (Clough and Bent, 1998). Transformed seedlings (T1) were identified by selection on solid MS medium containing kanamycin (50 mg L^−1^), and the plants were then transferred to soil. Further analysis of transgenic plants was carried out by PCR as described in Falcone Ferreyra et al. (2013) on the genomic DNA using the combination of primers: F-35S_prom_ and the reverse primer R-*RPL10A*-RT2. The expression of the *AtRPL10A* in transformed plants was analyzed by quantitative RT-PCR using F-*RPL10A*-RT2 and R-*RPL10A*-RT2 primers (Supplementary Table S1).

### Gene Expression Analyses by RT-qPCR

Total RNA was isolated from 100 mg of tissues using the TRIzol reagent (Invitrogen, Carlsbad, CA) followed by DNase treatment (Promega, Madison, WI). RNA was converted into first-strand cDNA using SuperScript II reverse transcriptase (Invitrogen) with oligo-dT as a primer. The resultant cDNA was used as a template for quantitative PCR amplification in a MiniOPTICON2 apparatus (Bio-Rad), using the intercalation dye SYBRGreen I (Invitrogen) as a fluorescent reporter and Platinum Taq DNA Polymerase (Invitrogen). Primers were designed to generate unique 150–250 bp fragments using the PRIMER3 software (Rozen and Skaletsky, 2000). The experiments were carried out using three biological replicates. Data of ABA treatments were normalized to *ACTIN2* (*ACT2*) transcript. All primer sequences are listed in Supplementary Table S1. Amplification conditions were as follows: 2 min denaturation at 94°C, 40–45 cycles at 94°C for 10 s, 57°C for 15 s, and 72°C for 20 s, followed by 10 min at 72°C. Melting curves for each PCR assay were determined by measuring the decrease of fluorescence with increasing temperature (from 65 to 98°C). To confirm the size of the PCR products and to check that they corresponded to a unique and expected product, the final products were separated on a 2% (w/v) agarose gel.

### Chlorophyll Extraction and Petiole Length Measurements

Total chlorophylls (Chl) were determined by standard procedures (Wintermans and De Mots, 1965). Leaves #3 were harvested, photographed, and petiole lengths were measured using ImageJ software.

### Primary Root Elongation and Lateral Root Measurements

Five-day-old seedlings grown on MS-agar plates were transferred to MS-agar plates supplemented or not with 10 μM ABA and held in vertical position in a growth chamber. Plates were photographed immediately after the transfer, and every day for the following 4 days. Images were analyzed using the ImageJ software. Experiments were performed in triplicate using 20 biological samples in each case.

### Stomatal Aperture Measurement and Water Loss Experiments

Fully expanded leaves #5 were harvested from 3-week-old wild type (WT Col-0), heterozygous *rpl10A-1* mutant plants, and *RPL10A*-overexpressing lines (*RPL10A*-OE3 and *RPL10A*-OE5) before the light period, placed into culture dishes and floated in an opening medium containing 10 mM KCl, 7.5 mM iminodiacetic acid, and 10 mM MES (pH 6.15) for 2.5 h in the growth chamber under dark to ensure that stomata were fully closed. Then, one group of samples were harvested, while the other group was transferred to the light and further incubated for 2 h in the same opening solution containing 20 μM ABA or without supplementation (control), to ensure that most of the stomata of the control samples were fully opened. After treatments (under dark and light conditions in the presence or absence of ABA), the abaxial epidermis was peeled from leaves and stomata were immediately photographed using a Leica microscope with a 40X objective. Stomatal apertures were measured using ImageJ software. For each condition, at least three leaves from different plants were used and 60 stomatal apertures were measured. Experiments were performed in triplicate.

For water loss measurement, fully expanded #5 leaves were detached from 3-week-old wild type (WT Col-0), heterozygous *rpl10A-1* mutant, and *RPL10A*-overexpressing plants, immediately weighed on a piece of weighing paper, and their petioles were immersed in a solution supplemented or not with 20 μM ABA under white light for 1 h. Leaves were further weighed and allowed to air dry for 5 h. Alternatively, 3-week-old rosettes of WT, heterozygous *rpl10A-1* mutant, and *RPL10A*-overexpressing plants were detached and water loss measurements were carried out as described above. Fresh weight was recorded at each hour and normalized to the initial fresh weight. The percentage of water loss (%) was calculated as the difference. Experiments were carried out in triplicate, using at least 10 leaves #5 from different plants in each experiment.

### Identification of Insertional T-DNA Heterozygous *rpl10A* Mutant Plants

For germination, early plant development and root elongation inhibition assays in the presence of ABA, heterozygous *rpl10A* mutant plants (*rpl10A/+*) were identified after the experiments were finished by PCR on genomic DNA using specific primers for *RPL10A* gene and one primer that hybridizes with the left border of the T-DNA. For stomatal aperture measurement, water loss experiments, and RT-qPCR analysis, *rpl10A-1/+* mutant plants were previously identified by PCR using genomic DNA extracted from leaves #1/#2 of 12-day-old seedlings. Primer sequences are listed in Supplementary Table S1.

### Statistical Analysis and *in silico* Analysis of RPL10A Promoter

Data were subjected to a two-factorial ANOVA, applying Tukey, Bonferroni, and Duncan tests (*p* < 0.05). When comparing two data sets, Student’s *t* test was used (*p* < 0.05). Statistical analysis of the data was obtained using InfoStat software v. 2017 (Di Rienzo et al., 2017).

*In silico* analysis of *RPL10A* promoter was done using the following database: AGRIS,^1^ AthaMap,^2^ PlantPAN2.0,^3^ and PLACE.^4^

### Accession Numbers

Sequence data from this article can be found in the *Arabidopsis* Genome Initiative under the following accession numbers: *ACTIN2*, At3g18780; *RPL10A*, At1g14320; *RPL10B*, At1g26910; and *RPL10C*, At1g66580.

## Results

### Expression of *RPL10A* During Germination and Early Seedling Establishment

Previous reported microarray and RNA-seq gene expression studies have indicated that *RPL10A* transcript levels increase at 6 h imbibition after stratification (Narsai et al., 2011) are maintained up to 24 h and are higher than *RPL10B* and *RPL10C* transcript levels (Klepikova et al., 2016; Supplementary Figures S1A,B). In addition, *RPL10A* transcript levels in root, cotyledons, hypocotyl, and shoot apical meristem from 1-day-old seedlings are higher than those of *RPL10B* and *RPL10C* (Klepikova et al., 2016; Supplementary Figure S1C). These comparative analyses show that *RPL10A* is the most abundantly expressed gene of the *RPL10* family members and suggest that RPL10A could play important roles during seed germination and early seedling development.

**Figure 1:**
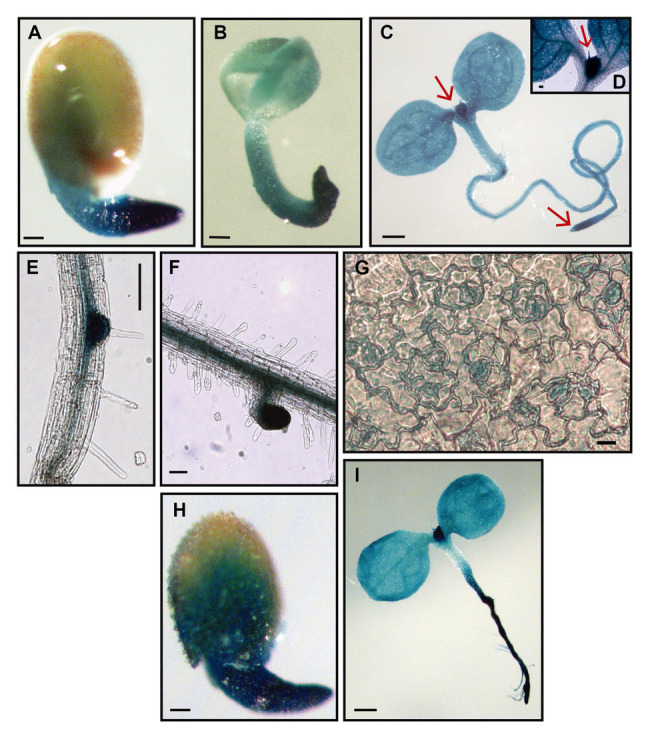
Histochemical GUS staining of transgenic *RPL10A_pro_:GUS* plants. **(A)** Germinated seed, 22 h after stratification. **(B)** Germinated seed without the testa, 22 h after stratification. **(C)** Four-day-old seedling at cotyledon greening stage. **(D)** Shoot apical meristem from 4-day-old seedling. **(E)** Primary root during lateral root emergence. **(F)** Lateral root from 7-day-old seedling. **(G)** Abaxial epidermis peeled from leaves #1/2 of 8-day-old seedlings. **(H)** Germinated seed, 46 h after stratification, with 1 μM abscisic acid (ABA). **(I)** Eight-day-old seedling at cotyledon greening stage, with 1 μM ABA. Arrows point to shoot apical meristem **(C,D)** and root apical meristem **(C)**. Scale bars, 100 μm **(A-C,H,I)** and 10 μm **(E-G)**.

Thus, we first analyzed *RPL10A* expression pattern during development using transgenic *RPL10A_pro_:GUS* plants previously generated (Falcone Ferreyra et al., 2013). *RPL10A* was highly expressed in germinated seeds (Figures 1A,B) and early seedlings (Figure 1C), with strong GUS staining in proliferating tissues such as shoot and root apical meristems (Figures 1C,D). Furthermore, high *RPL10A* expression was also observed during lateral root (LR) emergence and in elongated LRs (Figures 1E,F). GUS signal was also detected in stomatal cells (Figure 1G).

**Figure 2:**
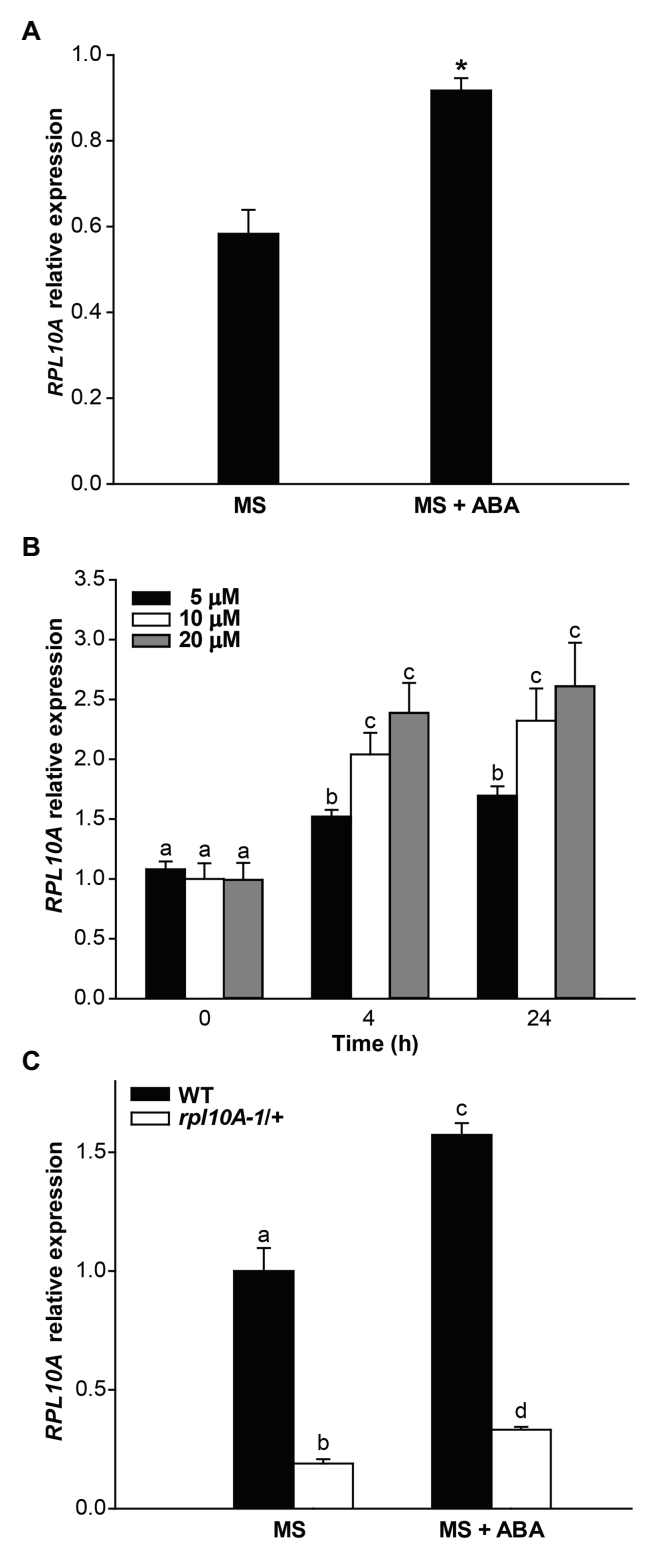
Regulation of the *RPL10A* expression by ABA. **(A)** *RPL10A* transcript levels in WT seedlings, at cotyledon greening stage, grown in MS-agar medium (MS) or in MS-agar medium with 1 μM ABA (MS + ABA), analyzed by RT-qPCR. Asterisks indicate statistical differences between conditions (Student’s *t*-test, *p* < 0.05). **(B)** *RPL10A* transcript accumulation in WT seedlings after ABA treatment (5, 10, and 20 μM) for 4 and 24 h. Values are expressed relative to the control set as 1. *ACTIN2* transcript accumulation was used as a reference. Results represent the average ± SE of three biological replicates. Different letters over the bar indicate statistical differences between samples applying ANOVA test (*p* < 0.05). **(C)** *RPL10A* transcript levels in WT and *rpl10A/+* mutant seedlings, at cotyledon greening stage, grown in MS-agar medium (MS) or in MS-agar medium with 1 μM ABA (MS + ABA). Values are expressed relative to the WT seedlings in MS-agar medium (MS) set as 1. *ACTIN2* transcript accumulation was used as a reference. Results represent the average ± SE of three biological replicates. Different letters over the bar indicate statistical differences between samples applying ANOVA test (*p* < 0.05).

To analyze whether RPL10A is involved in ABA responses, we determined the regulation of *RPL10A* expression by ABA treatment by RT-qPCR. Figure 2A shows that, at cotyledon greening stage (seedlings with fully open and green cotyledons), *RPL10A* expression is induced 1.6-fold by 1 μM ABA. This induction is observed as early as 4 h after the exposure of 14-day-old seedlings to 5, 10, and 20 μM ABA (Figure 2B). No differences in *RPL10A* transcript levels were observed between 10 and 20 μM ABA at 4 h or 24 h after treatment. To discard that other *RPL10* family genes can also be implicated in ABA responses, the expression of *RPL10B* and *RPL10C* genes was also analyzed by RT-qPCR. Transcript levels of both *RPL10B* and *RPL10C* were not modified by ABA treatment (Supplementary Figure S2A).

The induction of *RPL10A* expression by ABA was also examined using transgenic *RPL10A_pro_:GUS* plants. GUS activity was evaluated at germinated seed stage. Because ABA delays seed germination (Finkelstein et al., 2002; Yoshida et al., 2019), GUS activity was determined at 22 or 46 h after stratification in the absence (Figure 1A) or presence of 1 μM ABA (Figure 1H), respectively. Results show an increased GUS staining in the presence of ABA. GUS activity was also assessed at cotyledon greening stage. This stage was reached 4 and 8 days after stratification in the absence and presence of ABA, respectively. Although no appreciable differences in the GUS signal could be detected in the cotyledons of seedlings grown in the presence of ABA with respect to what was observed in its absence (Figure 1C), GUS staining markedly increased in the primary root by ABA (Figure 1I).

### RPL10A Is Involved in ABA-Mediated Seed Germination

**Figure 3:**
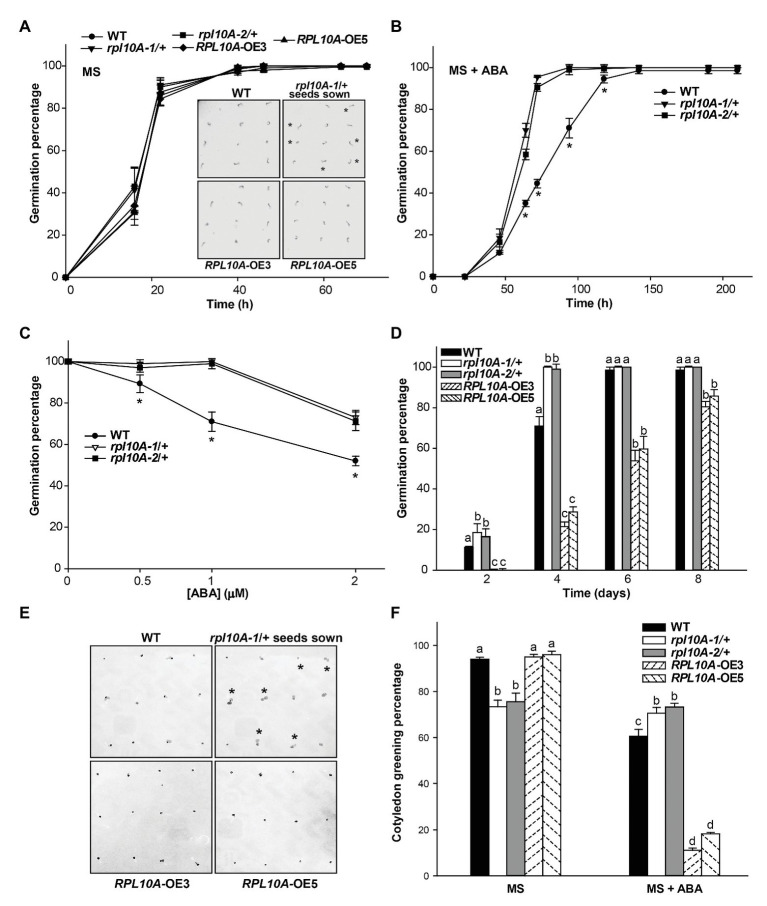
Effect of exogenous ABA on germination and cotyledon greening of *rpl10A/+* mutant and *RPL10A*-overexpressing seeds. **(A)** Germination percentage of wild type (WT), *rpl10A-1/+*, *rpl10A-2/+*, and *RPL10A*-overexpressing OE3 and OE5 seeds on MS-agar medium (MS). Inset: representative images of seedlings from all genotypes 1 day after stratification. Heterozygous *rpl10A-1* mutant seedlings are indicated with asterisks. **(B)** Germination percentage of WT and *rpl10A-1/+* and *rpl10A-2/+* seeds on MS-agar medium supplemented with 1 μM ABA (MS + ABA). Asterisks indicate statistical differences between WT and each mutant at each time applying Student’s *t*-test (*p* < 0.05). **(C)** ABA concentration-dependent germination assay. *rpl10A-1/+*, *rpl10A-2/+*, and WT seeds were germinated on MS-agar medium supplemented with increasing concentration of ABA and the germination percentages were scored at 4 days after stratification. Results represent the average of three independent experiments ± SE (*n* = 100 for each biological replicate). Asterisks indicate statistical differences between WT and each mutant at each time applying Student’s *t*-test (*p* < 0.05). **(D)** Germination percentage of WT, *rpl10A/+* mutants (*rpl10A-1/+* and *rpl10A-2/+*), and *RPL10A*-overexpressing seeds (*RPL10A*-OE3 and *RPL10A*-OE5) on MS-agar medium supplemented with 1 μM ABA at 2, 4, 6, and 8 days after stratification. Results represent the average of three independent experiments ± SE (*n* = 100 for each biological replicate). Different letters over the bars indicate statistical differences between genotypes at each time using ANOVA test (*p* < 0.05). **(E)** Representative images of WT, *rpl10A-1/+* mutants, and *RPL10A*-overexpressing lines (*RPL10A*-OE3 and *RPL10A*-OE5) grown on MS-agar medium supplemented with 1 μM ABA (MS + ABA), at 4 days after stratification. Heterozygous *rpl10A-1* mutant seedlings are indicated with asterisks. **(F)** Cotyledon greening percentage of WT, *rpl10A/+* mutants (*rpl10A-1/+* and *rpl10A-2/+*), and *RPL10A*-overexpressing lines (*RPL10A*-OE3 and *RPL10A*-OE5) grown on MS-agar medium (MS) or on MS-agar medium supplemented with 1 μM ABA (MS + ABA), at 8 days after stratification. Results are the mean of three independent experiments ± SE. For each genotype and condition (MS or MS + ABA), different letters over the bars indicate statistical differences applying ANOVA test (*p* < 0.05).

Germination of heterozygous *rpl10A* mutant (designed as *rpl10A-1/+* and *rpl10A-2/+*; Falcone Ferreyra et al., 2010a) and WT seeds was evaluated in MS-agar plates by scoring the radicle emergence. Fresh *rpl10A-1A+* and *rpl10A-2/+* mutant seeds showed a similar germination rate compared with WT seeds, reaching a maximum at 40 h after stratification (Figure 3A). However, in the presence of 1 μM ABA, *rpl10A/+* seeds showed an increased germination rate compared to WT seeds (Figure 3B). When percentage of inhibition of seed germination by ABA was compared between genotypes, *rpl10A/+* seeds exhibited lower values than WT seeds (Supplementary Figure S3A). The earlier germination pattern in *rpl10A/+* plants was also observed at 0.5 and 2 μM ABA (Figure 3C and Supplementary Figures S3B,C). To validate the role of RPL10A in ABA-mediated seed germination, *RPL10A*-overexpressing lines (*RPL10A*-OE3 and *RPL10A*-OE5) were generated. First, increased *RPL10A* transcript levels were confirmed by RT-qPCR in *RPL10A*-overexpressing lines (8.7-fold and 6.5-fold in *RPL10A*-OE3 and *RPL10A*-OE5, respectively, compared with those in WT plants; Supplementary Figure S2B). In addition, the expression of *RPL10B* and *RPL10C* genes was also analyzed in *RPL10A*-OE lines, and their transcript levels were similar to those in WT seedlings (Supplementary Figure S2B). Germination of *RPL10A*-OE lines was further analyzed. Germination of *RPL10A*-OE lines was similar to that of WT seeds in the absence of ABA (Figure 3A and inset). However, in the presence of exogenous applied ABA (1 μM), *RPL10A*-OE3 and *RPL10A*-OE5 lines were more sensitive to ABA than WT seeds, showing significantly reduced germination rates compared to WT seeds at 4 days after stratification (Figures 3D,E). These results suggest that RPL10A is required for the inhibition of seed germination by ABA.

### *rpl10A/+* Mutant Plants Show Reduced Sensitivity to ABA During Early Development

Then, we investigated ABA sensitivity of *rpl10A/+* mutants and transgenic *RPL10A* overexpressing lines during early plant development. The addition of exogenous ABA decreased the cotyledon greening percentage to 60, 11, and 18% of the untreated control in WT, *RPL10A*-OE3, and *RPL10A*-OE5 lines, respectively (Figure 3F). Supplementary Figure S4A shows representative images of seedlings from WT, mutant, and overexpressing genotypes at 4 and 8 days after stratification in the absence and presence of ABA, respectively. It should be mentioned that under control conditions *rpl10A-1/+* and *rpl10A-2/+* mutant plants only exhibited 73 and 75% cotyledon greening at 8 days after stratification, respectively; whereas *RPL10A*-OE3 and *RPL10A*-OE5 lines showed maximum and similar percentages (about 98%) to those observed in WT plants (Figure 3F). However, *rpl10A/+* mutant plants did not show a decrease in cotyledon greening percentage in response to ABA treatment respect to the control condition, exhibiting similar percentages under both growth conditions (Figure 3F). Thus, we also investigated seedling establishment of *rpl10A/*+ mutants after cotyledon greening stage. Consistently, *rpl10A/*+ seeds showed a deficient juvenile vegetative development (68 and 71% for *rpl10A-1/+* and *rpl10A-2/+*, respectively) compared to WT seeds (94%) at 12 days after stratification in MS-agar control medium (Supplementary Figures S4B,C). Several mutant seedlings arrested their growth before the emergence or expansion of true leaves and died, suggesting that RPL10A is necessary for this developmental stage. In contrast, transgenic *RPL10A*-overexpressing lines showed no differences in seedling development with respect to WT.

**Figure 4:**
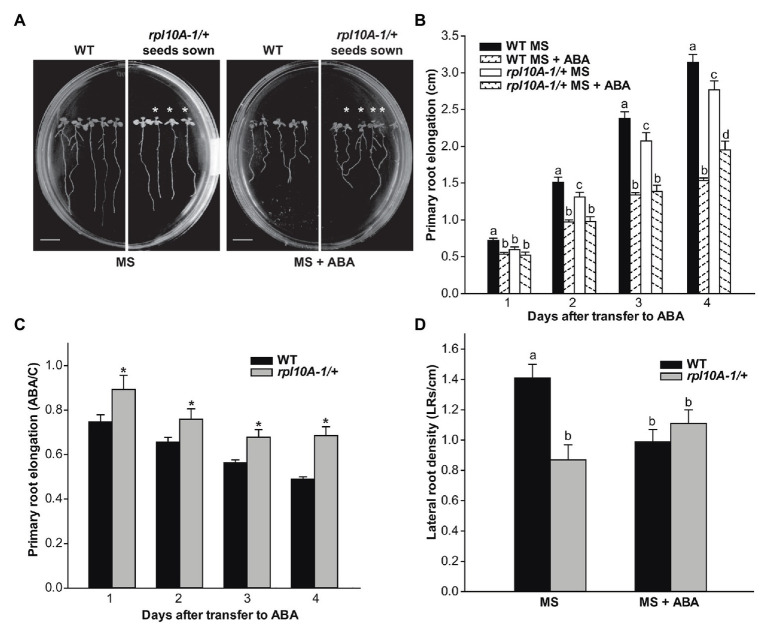
ABA inhibition of primary root elongation and lateral root development of WT and *rpl10A/+* mutant seedlings. **(A)** Representative images of WT and *rpl10A-1/+* mutant seedlings grown on MS-agar plates and transferred to MS-agar medium (MS) or MS-agar medium supplemented with 10 μM ABA (MS + ABA) 4 days after the transfer. Heterozygous *rpl10A-1* mutant seedlings are indicated with asterisks. Bars = 1 cm. **(B)** Primary root elongation of WT and *rpl10A-1/+* mutant seedlings measured each day after the transfer to MS-agar medium (MS) or MS-agar medium supplemented with 10 μM ABA (MS + ABA). Results are the mean of three independent experiments (*n* = 20 for each biological experiment). For each genotype and condition (MS or MS + ABA), different letters over the bars indicate statistical differences applying ANOVA test (*p* < 0.05). **(C)** Average root length after the transfer to ABA treatment relative to control conditions (ABA/C). Asterisks indicate statistical differences between genotype at each day (Student’s *t*-test, *p* < 0.05). **(D)** LR density estimated as the number of LRs per root length unit of WT and *rpl10A-1/+* mutants. The number of LRs was determined 4 days after the transfer of 5-day-old seedlings to plates supplemented or not with ABA. Results are the mean of three independent experiments ± SE (*n* = 20 for each biological experiment). For each genotype and condition (MS or MS + ABA), different letters over the bars indicate statistical differences applying ANOVA test (*p* < 0.05).

Root growth in response to ABA was also evaluated. Five-day-old WT and *rpl10A-1/+* mutant seedlings were transferred from MS-agar control medium to new plates, either containing MS medium supplemented or not with 10 μM ABA. Figure 4A shows representative images of seedlings from both genotypes 4 days after treatment. In the absence of ABA, *rpl10A-1/+* mutants showed shorter primary roots and a lower number of LRs per root length unit (LRs density) than WT plants (Figures 4B–D). In the presence of ABA, both WT and *rpl10A-1/+* mutants showed primary root elongation inhibition, but the inhibition by ABA was lower in *rpl10A-1/+* mutants than in WT plants at 4 days after the transfer (Figures 4B,C), demonstrating that *rpl10A-1/+* plants show a lower sensitivity to ABA than WT plants. Accordingly, *rpl10A-1/+* mutants displayed no inhibition of LR development in response to exogenous ABA (Figure 4D), while the LRs density was decreased in WT plants (Figure 4D).

Primary root elongation was also investigated in *RPL10A*-OE plants. Under control conditions, *RPL10A*-OE lines had longer primary roots with more LRs per root length unit than WT plants (Supplementary Figures S5A–D). However, primary root elongation and LR development were inhibited to the same extent by ABA treatment in overexpressing and WT plants (Supplementary Figures S5C,E), suggesting that a threshold RPL10A level seems to be enough for its root elongation function.

**Figure 5:**
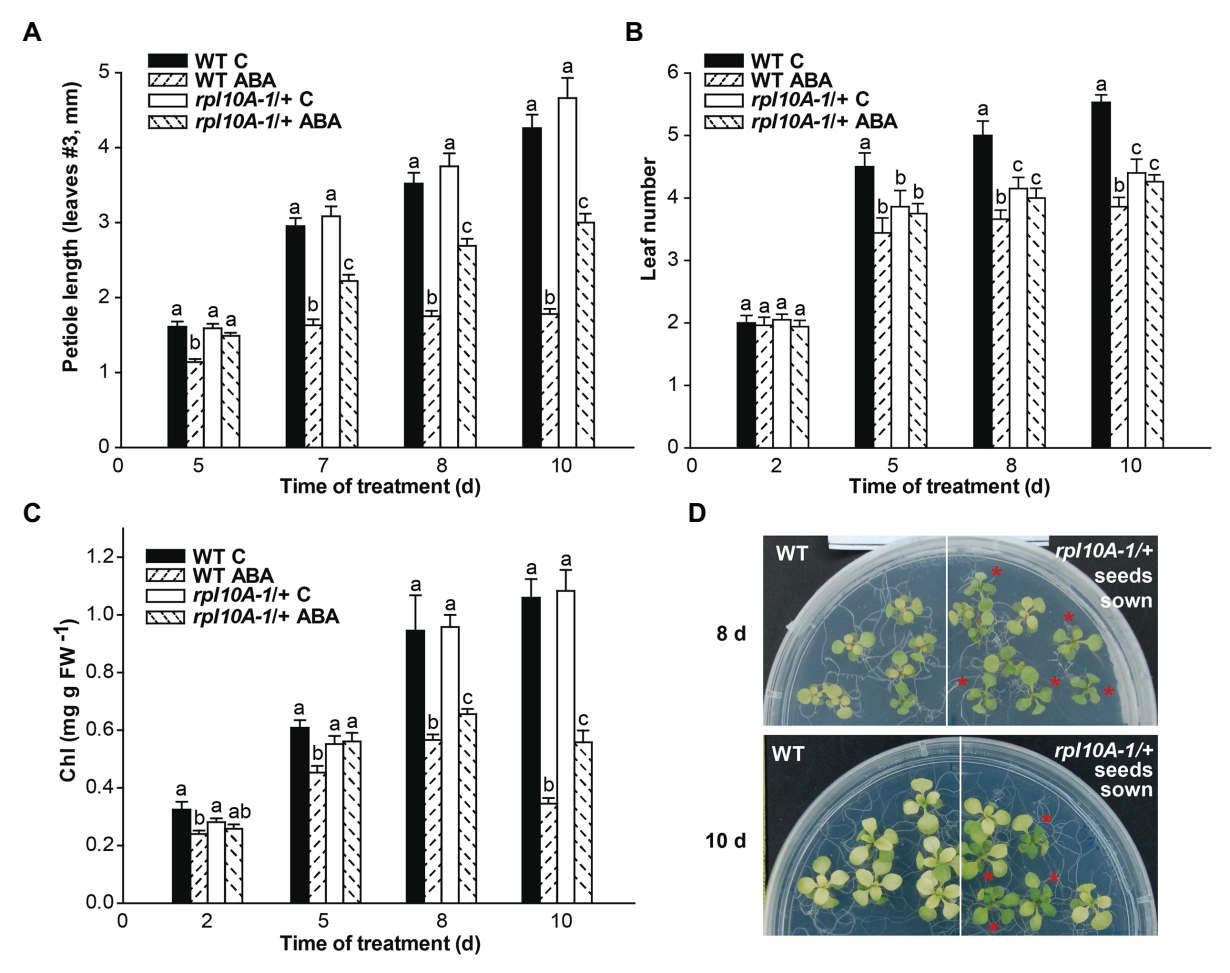
ABA sensitivity time course of *rpl10A/+* seedlings. Seven-day-old seedlings grown in MS-agar medium were transferred to MS-agar (MS) or MS-agar medium supplemented with 10 μM ABA (MS + ABA). Petiole length **(A)**, leaf number **(B)**, and chlorophyll (Chl) content per mg fresh weight^−1^ (FW; **C**) were measured over the time for 10 days. **(D)** Representative images of WT and *rpl10A-1/+* mutant plants at 8 days (upper panel) and 10 days after the transfer to MS-agar medium supplemented with 10 μM ABA (lower panel). Asterisks indicate heterozygous *rpl10A-1* mutant seedlings. Results are the mean of three independent experiments ± SE (*n* = 30 for each biological experiment). For each genotype and condition (with or without 10 μM ABA), different letters indicate statistical differences applying ANOVA test at *p* < 0.05.

We also determined the effect of ABA on petiole length, leaf number, and yellowing (Figure 5). WT plants treated with ABA (10 μM) were found to have shorter petioles of leaf #3 (Figure 5A), fewer developed leaves (Figure 5B), and increased leaf yellowing as determined by the amount of total chlorophyll (Figure 5C) compared with the control analyzed over the time of the treatment after the transfer to MS-agar medium supplemented with ABA. Notably, *rpl10A-1/+* mutants exhibited less ABA sensitivity than WT plants, showing a lower reduction of petiole length (Figure 5A) and yellowing (Figure 5C) than WT plants, while the leaf number was not modified by ABA treatment (Figure 5B). Figure 5D shows representative images of plants from both genotypes at 8 days (upper panel) and at 10 days (lower panel) after the transfer. Similar results were observed when the treatment was carried out with 5 μM ABA (Supplementary Figure S6).

### ABA-Regulated Stomatal Movement and Water Loss Are Impaired in *rpl10A* Mutants

**Figure 6:**
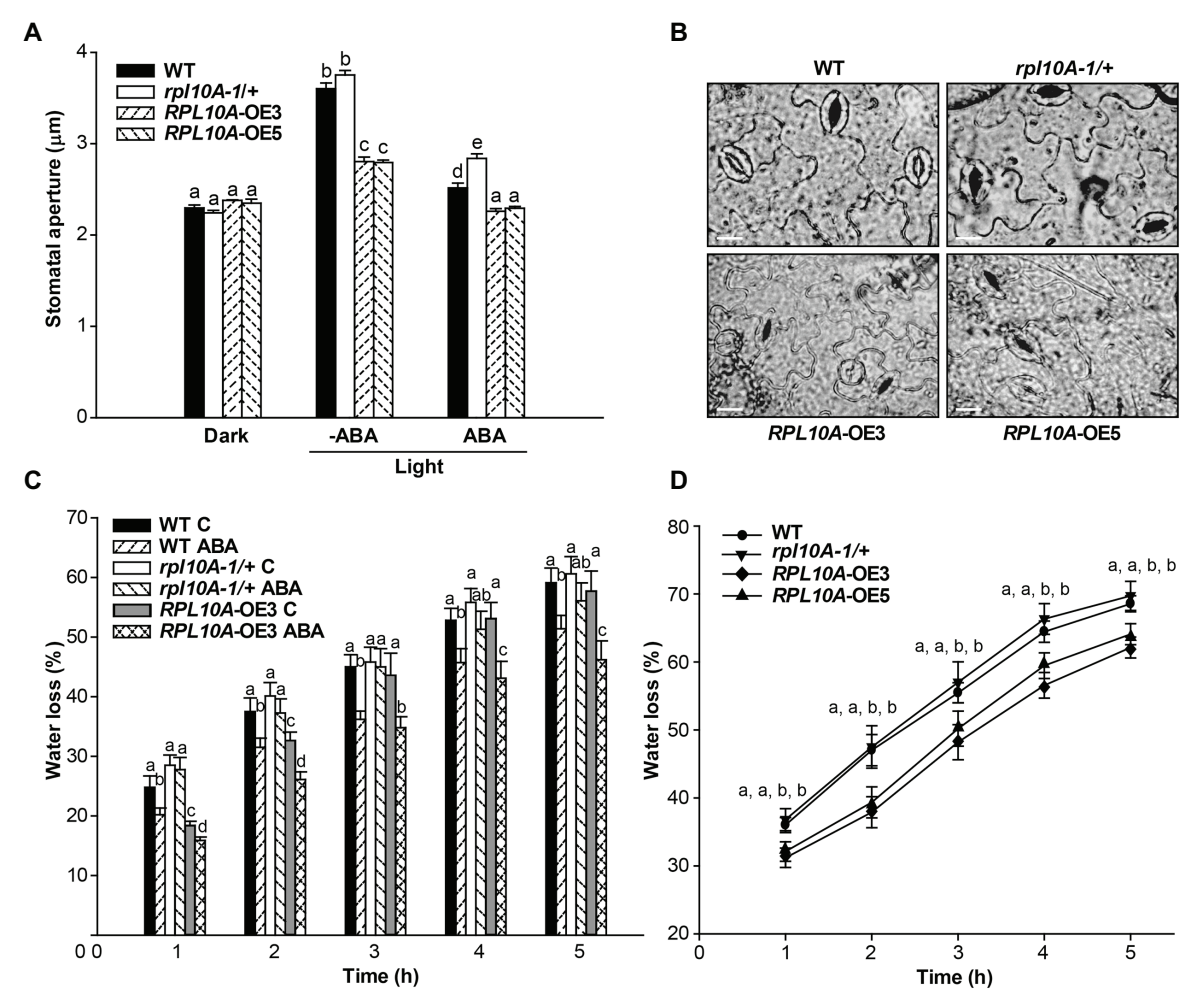
Effect of ABA on stomatal aperture and water loss. **(A)** Stomatal aperture was measured on epidermal peels of leaves #5 from WT, *rpl10A-1/+* mutants, and *RPL10A*-overexpressing lines (*RPL10A*-OE3 and *RPL10A*-OE5) plants after 2 h in the dark and subsequent incubation without (−ABA) or with 20 μM ABA (+ABA) under white light for 2 additional hour. Results are the mean of three independent experiments ± SE For each experiment, at least three leaves from three different plants were used and 60 stomata were analyzed. For each genotype and condition (dark and light with or without 20 μM ABA), different letters over the bars indicate significant differences applying ANOVA test at *p* < 0.05. **(B)** Representative images of abaxial epidermis peeled from leaves #5 of 3-week-old WT, *rpl10A-1/+* mutants, and *RPL10A*-overexpressing lines (*RPL10A*-OE3 and *RPL10A*-OE5) plants incubated with 20 μM ABA under white light for 2 h. Bar = 10 μm. **(C)** Water loss of air-dried leaves #5 from 3-week-old WT, *rpl10A/+* mutant and *RPL10A*-OE3 plants after petiole-feeding with 20 μM ABA or water (Control, **C**) for 1 h under white light. Fresh weight was measured every hour and data were normalized to the initial fresh weight. Results represent the average of three independent experiments ± SE, *n* = 10 for each biological replicate. At each time, for each genotype and condition (with or without 20 μM ABA), different letters over the bars indicate statistical differences applying ANOVA test (*p* < 0.05). **(D)** Water loss of air-dried rosettes from 3-week-old WT, *rpl10A-1/+* mutant, and *RPL10A*-overexpressing plants (*RPL10A*-OE3 and *RPL10A*-OE5) maintained under control conditions. Fresh weight was measured every hour and data were normalized to the initial fresh weight. Results represent the average of three independent experiments ± SE, *n* = 10 for each biological replicate. Different letters indicate statistical differences between genotypes at each time applying ANOVA test at *p* < 0.05.

Public microarray data show that *RPL10A* transcript levels increased 1.3-fold and 1.4-fold in guard and mesophyll cells exposed to ABA, respectively (Supplementary Figure S1D; Winter et al., 2007), and here we show that *RPL10A* is expressed in stomatal cells (Figure 1G). To investigate if ABA-mediated stomatal movement is affected in *rpl10A* mutant and *RPL10A*-overexpressing plants, stomata were induced to open under white light in the presence and absence of 20 μM ABA. No differences in stomatal apertures in dark-adapted leaves in the absence of ABA were observed between genotypes. In illuminated leaves, WT and *rpl10A/+* mutant plants did not show differences in stomatal apertures, but *RPL10A*-overexpressing plants showed less stomatal opening compared to WT plants. ABA treatment caused a decrease of stomatal opening in all plants, but *rpl10A-1/+* mutants were less responsive to ABA-promoted stomatal closure than WT plants, while *RPL10A*-OE lines exhibited the most decrease in this parameter (Figure 6A). Figure 6B shows representative images of stomata from abaxial epidermis of leaf #5 from all genotypes treated with 20 μM ABA. We also evaluated the kinetics of water loss. Treatment with 20 μM ABA leads *rpl10A-1/+* mutants to lose water faster than WT plants (Figure 6C), probably due to their less sensitivity to ABA-induced stomatal closure, suggesting that RPL10A could be a player in the regulation of guard cell ABA signaling. On the contrary, leaves #5 of *RPL10A*-OE3 line showed less water loss than WT plants, both under control conditions and after ABA treatment (Figure 6C). In 3-week-old whole rosettes maintained under control conditions, similar results were observed for *RPL10A*-OE3 and *RPL10A*-OE5 lines, while *rpl10A-1/+* mutants showed similar water loss to WT plants without ABA treatment (Figure 6D).

### Transcriptional Responses of *RPL10A* Gene to ABA

**Figure 7:**
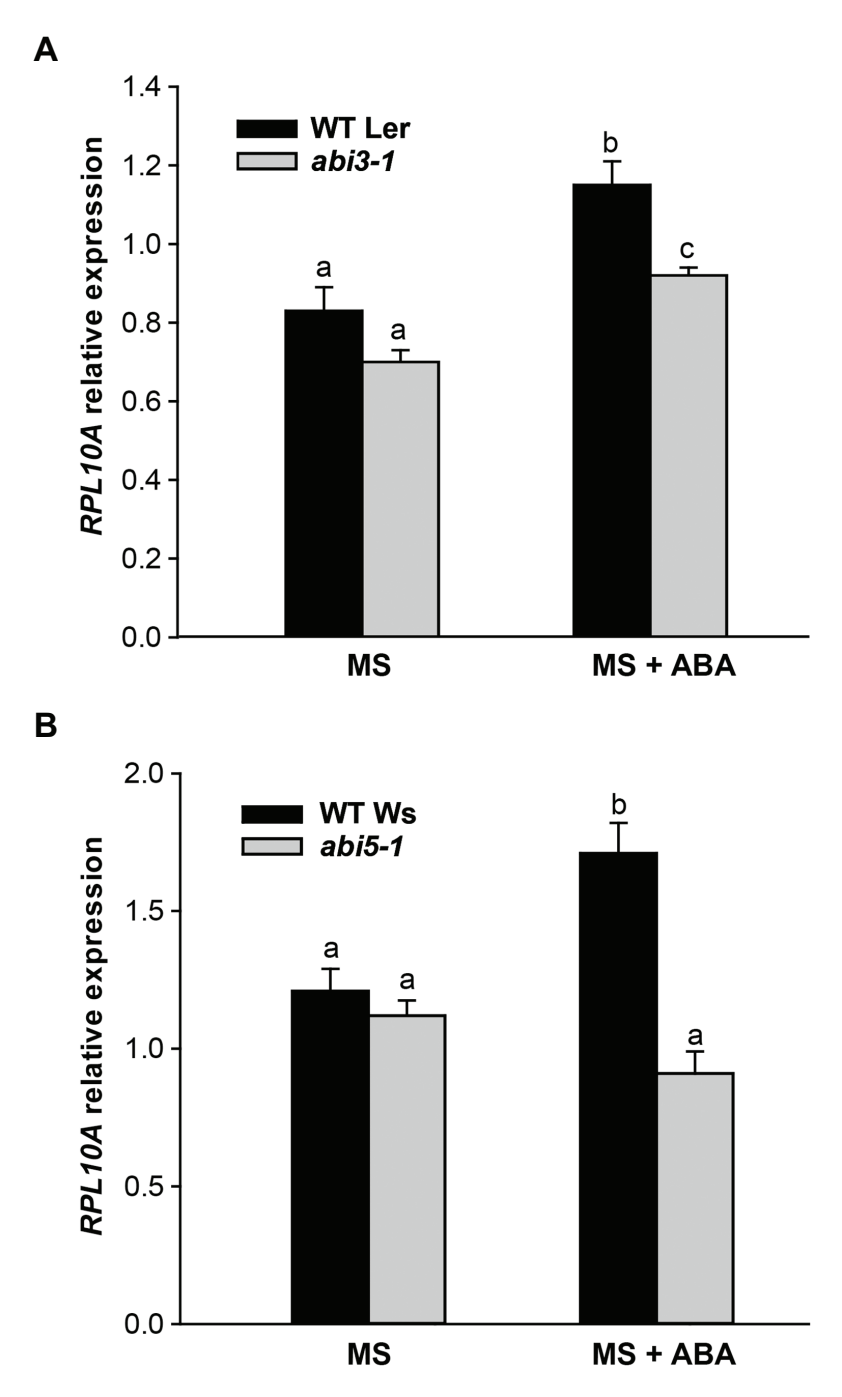
*RPL10A* expression analysis in *abi3* and *abi5* mutant plants. *RPL10A* transcript levels in *abi3-1* **(A)** and *abi5-1* **(B)** mutant plants and their respective WT background plants (Ler and Ws, respectively) grown in MS-agar medium without (MS) or with 1 μM ABA supplementation (MS + ABA). *ACTIN2* transcript accumulation was used as a reference. Results represent the average ± SE of three biological replicates. Different letters over the bar indicate statistical differences between samples applying ANOVA test (*p* < 0.05).

Next, we investigated whether the induction of *RPL10A* by ABA depended on *ABI3* and *ABI5* transcription factors. Figures 7A,B show that *RPL10A* transcript levels did not increase in *abi3* and *abi5* mutants as in WT seedlings. In the absence of ABA, *RPL10A* expression was similar in all genotypes.

We further analyzed if a decreased sensitivity to ABA of *rpl10A/+* mutants could be related to an altered expression of *RPL10* genes. Fourteen-day-old WT seedlings and *rpl10A-1/+* mutants previously grown in MS-agar plates were transferred to liquid MS medium with or without ABA supplementation and further incubated under light conditions for 4 h. In *rpl10A-1/+* heterozygous mutant plants, the induction of *RPL10A* expression by ABA was similar to the one observed in WT plants, but transcript levels of mutant plants were significantly lower than those in WT plants after treatment (Supplementary Figure S2A). Moreover, to confirm the absence of functional compensation in the RPL10 family by variations in their transcript levels, the expression of *RPL10B* and *RPL10C* genes was analyzed by RT-qPCR in *rpl10A-1/+* mutants treated with ABA. Transcript levels of *RPL10B* and *RPL10C* were similar in seedlings under control and ABA condition (Supplementary Figure S2A). In addition, at cotyledon greening stage (seedlings with fully open and green cotyledons), *RPL10A* expression is induced in *rpl10A-1/+* mutants (1.7-fold) as in WT plants (1.6-fold) by 1 μM ABA (Figure 2C). Thus, both the regulation of *RPL10A* expression by ABA and the expression of other *RPL10* family members are not altered in *rpl10A/+* mutant plants.

## Discussion

Many studies have shown that distinct paralogous family members of ribosomal proteins could play a role in different developmental stages/tissues, under specific stress conditions (Byrne, 2009; Horiguchi et al., 2012; Hummel et al., 2012; Carroll, 2013), or in response to hormones (Byrne, 2009; Rosado et al., 2012). Besides their roles in translation, ribosomal proteins have also been reported to have extra-ribosomal functions. Recently, an extra-ribosomal function in miRNA biogenesis was attributed to *A. thaliana* RPL24B (Li et al., 2017). Previously, RPL10A was demonstrated to be essential for plant viability and also to play roles in abiotic and biotic stress responses in *A. thaliana* and rice (Falcone Ferreyra et al., 2010a,b, 2013; Zorzatto et al., 2015; Moin et al., 2016a). Under some of these stresses, the protein could be detected both in cytosol and nuclei, thus suggesting extra-ribosomal functions (Falcone Ferreyra et al., 2013). In addition, ribosomal proteins have been involved in modulating gene expression (Merchante et al., 2017; Calamita et al., 2018; Genuth and Barna, 2018). Specifically, RPL10A was reported to regulate gene expression both at a transcriptional level, acting as a transcriptional repressor, and at a translational level, as a component of the 80S ribosome (Falcone Ferreyra et al., 2010a; Zorzatto et al., 2015). One demonstrated step that regulates gene expression is the upstream open reading frame (uORF)-mediated translational control (Merchante et al., 2017). In fact, RPL10A has been demonstrated to control uORF-mediated translation (Imai et al., 2008). In this work, we extend these studies by analyzing the additional roles of RPL10A in *A. thaliana*.

We first analyzed the spatial and temporal expression of *RPL10A*. The gene exhibits a ubiquitous expression pattern throughout the plant, but it is most strongly expressed in undifferentiated tissues with active division, in primary and LRs, both at emergency and at outgrowth stages and in elongated LRs (Figure 1). Notably, gene expression was also detected in stomatal cells (Figure 1). These data are supported by microarray data of different *Arabidopsis* tissues (Supplementary Figure S1). Supplementary Figure S1 shows high *RPL10A* transcript levels in all types of cells, being the highest in the shoot apical meristem, but high levels of expression are also present in roots and cotyledons.

We then examined the role of RPL10A during early plant development and ABA response using *Arabidopsis rpl10A* heterozygous mutants and *RPL10A*-overexpressing lines. Under control conditions, no differences during seed germination (Figure 3A) were observed between genotypes. However, *rpl10A/+* mutants showed reduced cotyledon greening (Figure 3F) and decreased early seedling development (Supplementary Figure S4) compared to WT plants. In addition, *rpl10A/+* mutant seedlings showed defects in root growth, with shorter primary roots and impaired LR formation compared to WT seedlings (Figure 4). In contrast, although *RPL10A*-overexpressing lines did not exhibit differences in cotyledon greening and seedling development respect to WT plants, they showed longer primary roots with higher LRs density than WT plants (Figure 3; Supplementary Figures S4, S5). These results may indicate that a RPL10A threshold level may be necessary for plant development specially during the early stages when active cell division occurs, possibly attributed to its key function to form mature 80S ribosomes during protein synthesis, as it was previously reported for this protein and other *Arabidopsis* ribosomal proteins (Degenhardt and Bonham-Smith, 2008; Byrne, 2009; Falcone Ferreyra et al., 2010a). Accordingly, public RNA-Seq data of *Arabidopsis* WT cells after light exposition over time show an increase in *RPL10A* transcript levels (Petryszak et al., 2016) supporting the RPL10A involvement in seed-to-seedling transition. Additionally, protein-protein interaction networks constructed using AraPPINet (http://netbio.sjtu.edu.cn/arappinet/; Zhang et al., 2016) show that a large group of possible protein interactors with RPL10A are involved in photomorphogenesis and plant growth and development. These findings indicate that RPL10A may also have roles in these physiological processes and, consequently, it could explain the growth arrest of germinated seeds in *rpl10A/+* mutants.

ABA was reported to negatively regulate seed germination and plant development (Finkelstein et al., 2002; Yoshida et al., 2019) and to control primary and LR development (Santos Teixeira and ten Tusscher, 2019). It was also demonstrated that ribosomal proteins can play roles in ABA responses through their participation in protein translation (Wang et al., 2011). Consequently, we investigated if RPL10A is involved in ABA response during seed germination and seedling development. First, we observed that *RPL10A* expression is induced by ABA (Figures 1H,I, 2A,B). This result is in accordance with previous reported data (Supplementary Figure S1D; Petryszak et al., 2016). We also showed that in the presence of exogenous ABA, *rpl10A/+* seeds germinated earlier than WT seeds, while the opposite was observed in *RPL10A*-OE lines (Figures 3B–E). *rpl10A/+* mutants also showed less ABA-inhibition of cotyledon greening, primary root elongation, LR formation, petiole length, leaf number, and chlorophyll content compared to WT seedlings (Figures 3–5). Similarly, *Arabidopsis* mutant plants in *RACK1A*, a protein with many different functions, which include translation, development, ABA-signaling, and auxin-signaling, showed insensitivity to LR development inhibition by salinity, an abiotic stress that involved ABA action (Denver and Ullah, 2019). Thus, based on these data and the phenotypes observed in this work, RPL10A seems to be a positive regulator of ABA responses.

Because ABA inhibits stomatal opening under light conditions (Kim et al., 2010; Assmann and Jegla, 2016), we also investigated the RPL10A role in stomatal aperture. Both leaves #5 and detached rosettes of *RPL10A*-overexpressing lines lost significantly less water than WT in correlation with a high stomatal closure observed under light condition both with and without ABA treatment (Figure 6). On the contrary, *rpl10A/+* mutant plants did not exhibit differences with respect to WT in water loss of both leaf #5 and detached rosettes under control condition but showed a decreased ABA sensitivity in guard cells, with a higher stomatal aperture under light conditions and an increased water loss after the treatment (Figure 6). These results are consistent with the *in vivo* localization of RPL10A in guard cells (Figure 1G). Even more, protein-protein interaction networks predict that RPL10A interacts with proteins that regulate the stomatal closure, such as ATH-BTB domain proteins (BPMs) and the negative regulator of ABA response, the class I homeobox-leucine zipper (HD-ZIP) transcription factor ATHB6 (Lechner et al., 2011). Consequently, the involvement of RPL10A in these physiological processes in response to ABA could be mediated through its interaction with these and other proteins involved in stomatal movement. In fact, previous co-immunoprecipitation studies showed that RPL10A interacts with TGG1 and TGG2 myrosinases, both proteins involved in ABA signaling in guard cells (Islam et al., 2009; Falcone Ferreyra et al., 2010a,b).

To investigate a link between RPL10A and ABA-dependent responses, we analyzed *RPL10A* induction by ABA. This induction is lower or does not take place in *abi3-1* and *abi5-1* mutants, respectively (Figures 7A,B), suggesting that *RPL10A* expression might be regulated by *ABI3* and *ABI5* transcription factors. *In silico* analysis of *RPL10A* promoter show the presence of cis-regulatory elements associated with hormone regulation, stress, and development (Supplementary Figure S7). For example, G-box elements, involved in ABA responses, binding sites for bZIP transcription factors (TFs) such as ABI5 (Finkelstein and Lynch, 2000) and RY elements for B3-type TFs such as ABI3, among others are predicted in the *RPL10A* promoter. ABA signaling also involves diverse types of transcription factors, such as NACs, MYCs, MYBs, ERFs (ethylene response factors), and WRKYs (Liu et al., 2012; Sah et al., 2016), some of which were also predicted in the *RPL10A* promoter. It is then possible that *RPL10A* regulation by ABA can involve multiple TFs and more than one signaling pathway.

Taken together, these results indicate that RPL10A could play roles in ABA-dependent responses at one or more different levels. One possibility is that nuclear RPL10A can transcriptionally regulate the expression of genes involved in ABA signaling and/or be involved in the post-transcriptional regulation through its binding to RNA or RNA-binding proteins. Accordingly, RPL10A co-immunoprecipitated proteins involved in mRNA stability such as the glycine-rich protein GRP7 (Falcone Ferreyra et al., 2010a) and protein-protein interaction networks predict that RPL10A can interact with a RNA-binding proteins proposed to be involved in exon junction and in RNA processing. In addition, RPL10A may have a precise role in protein synthesis of key components of ABA responses. This hypothesis, focus of future studies, agrees with our data showing that *RPL10A* is induced by ABA and with previous reports indicating that this gene is translational regulated and that a reconfiguration of translatomes by different stress can occur to facilitate acclimation (Mustroph et al., 2009; Falcone Ferreyra et al., 2010a). Our results deserve further analysis by RNA-seq technology to provide insights about WT, mutant, and transgenic plant response to ABA treatment and to characterize regulatory gene networks. Future work is needed to investigate how RPL10A is involved in ABA signaling.

## Data Availability Statement

The raw data supporting the conclusions of this article will be made available by the authors, without undue reservation.

## Author Contributions

MLFF designed the study. RR performed the experiments. MLFF, RR, CS, and PC analyzed and interpreted the data. All authors contributed to the writing of the manuscript and approved the final version.

### Conflict of Interest

The authors declare that the research was conducted in the absence of any commercial or financial relationships that could be construed as a potential conflict of interest.

## Footnotes

^1^https://agris-knowledgebase.org/AtcisDB

^2^http://www.athamap.de

^3^http://plantpan2.itps.ncku.edu.tw

^4^https://www.dna.affrc.go.jp/PLACE/?action=newplace

## References

[ref1] Assmann S. M. Jegla T. (2016). Guard cell sensory systems: recent insights on stomatal responses to light, abscisic acid, and CO2. Curr. Opin. Plant Biol. 33, 157–167. 10.1016/j.pbi.2016.07.003, PMID: 27518594

[ref2] Basbouss-Serhal I. Soubigou-Taconnat L. Bailly C. Leymarie J. (2015). Germination potential of dormant and nondormant *Arabidopsis* seeds is driven by distinct recruitment of messenger RNAs to polysomes. Plant Physiol. 168, 1049–1065. 10.1104/pp.15.00510, PMID: 26019300 PMC4741348

[ref3] Byrne M. E. (2009). A role for the ribosome in development. Trends Plant Sci. 14, 512–519. 10.1016/j.tplants.2009.06.009, PMID: 19716746

[ref4] Calamita P. Gatti G. Miluzio A. Scagliola A. Biffo S. (2018). Translating the game: ribosomes as active players. Front. Genet. 9:533. 10.3389/fgene.2018.00533, PMID: 30498507 PMC6249331

[ref5] Carroll A. J. (2013). The *Arabidopsis* cytosolic ribosomal proteome: from form to function. Front. Plant Sci. 4:32. 10.3389/fpls.2013.00032, PMID: 23459595 PMC3585428

[ref6] Cherepneva G. N. Schmidt K. H. Kulaeva O. N. Oelmüller R. Kusnetsov V. V. (2003). Expression of the ribosomal proteins S14, S16, L13a and L30 is regulated by cytokinin and abscisic acid: implication of the involvement of phytohormones in translational processes. Plant Sci. 165, 925–932. 10.1016/S0168-9452(03)00204-8

[ref7] Clough S. J. Bent A. F. (1998). Floral dip: a simplified method for agrobacterium-mediated transformation of *Arabidopsis thaliana*. Plant J. 16, 735–743. 10.1046/j.1365-313X.1998.00343.x, PMID: 10069079

[ref8] Degenhardt R. F. Bonham-Smith P. C. (2008). *Arabidopsis* ribosomal proteins RPL23aA and RPL23aB are differentially targeted to the nucleolus and are disparately required for normal development. Plant Physiol. 147, 128–142. 10.1104/pp.107.111799 18322146 PMC2330296

[ref9] Denver J. B. Ullah H. (2019). miR393s regulate salt stress response pathway in *Arabidopsis thaliana* through scaffold protein RACK1A mediated ABA signaling pathways. Plant Signal. Behav. 14, 1–7. 10.1080/15592324.2019.1600394, PMID: 31021701 PMC6546147

[ref500] Di Rienzo J. A. Casanoves F. Balzarini M. G. Gonzalez L. Tablada M. Robledo C. V. (2017). InfoStat versión 2017. Grupo InfoStat, FCA, Universidad Nacional de Córdoba, Argentina.28327617

[ref10] Falcone Ferreyra M. L. Biarc J. Burlingame A. L. Casati P. (2010b). *Arabidopsis* l10 ribosomal proteins in UV-B responses. Plant Signal. Behav. 5, 1222–1225. 10.4161/psb.5.10.12758, PMID: 20855946 PMC3115351

[ref11] Falcone Ferreyra M. L. Casadevall R. Luciani M. D. Pezza A. Casati P. (2013). New evidence for differential roles of L10 ribosomal proteins from *Arabidopsis*. Plant Physiol. 163, 378–391. 10.1104/pp.113.223222, PMID: 23886624 PMC3762657

[ref12] Falcone Ferreyra M. Pezza A. Biarc J. Burlingame A. L. Casati P. (2010a). Plant L10 ribosomal proteins have different roles during development and translation under ultraviolet-B stress. Plant Physiol. 153, 878–1894. 10.1104/pp.110.157057, PMID: 20516338 PMC2923885

[ref13] Finkelstein R. R. Gampala S. S. L. Rock C. D. (2002). Abscisic acid signaling in seeds and seedlings. Plant Cell 14, 15–46. 10.1105/tpc.010441, PMID: 12045268 PMC151246

[ref14] Finkelstein R. R. Lynch T. J. (2000). The *Arabidopsis* abscisic acid response gene ABI5 encodes a basic leucine zipper transcription factor. Plant Cell 12, 599–609. 10.1105/tpc.12.4.599, PMID: 10760247 PMC139856

[ref15] Genuth N. R. Barna M. (2018). The discovery of ribosome heterogeneity and its implications for gene regulation and organismal life. Mol. Cell 71, 364–374. 10.1016/j.molcel.2018.07.018, PMID: 30075139 PMC6092941

[ref16] Guo J. Wang S. Valerius O. Hall H. Zeng Q. Li J. F.. (2011). Involvement of *Arabidopsis* RACK1 in protein translation and its regulation by abscisic acid. Plant Physiol. 155, 370–383. 10.1104/pp.110.160663, PMID: 21098678 PMC3075769

[ref17] Hofer A. Bussiere C. Johnson A. W. (2007). Mutational analysis of the ribosomal protein Rpl10 from yeast. J. Biol. Chem. 282, 32630–32639. 10.1074/jbc.M705057200, PMID: 17761675

[ref18] Horiguchi G. Van Lijsebettens M. Candela H. Micol J. L. Tsukaya H. (2012). Ribosomes and translation in plant developmental control. Plant Sci. 191–192, 24–34. 10.1016/j.plantsci.2012.04.008, PMID: 22682562

[ref19] Hummel M. Cordewener J. H. G. de Groot J. C. M. Smeekens S. America A. H. P. Hanson J. (2012). Dynamic protein composition of *Arabidopsis thaliana* cytosolic ribosomes in response to sucrose feeding as revealed by label free MSE proteomics. Proteomics 12, 1024–1038. 10.1002/pmic.201100413, PMID: 22522809

[ref20] Imai A. Komura M. Kawano E. Kuwashiro Y. Takahashi T. (2008). A semi-dominant mutation in the ribosomal protein L10 gene suppresses the dwarf phenotype of the acl5 mutant in *Arabidopsis thaliana*. Plant J. 56, 881–890. 10.1111/j.1365-313X.2008.03647.x, PMID: 18694459

[ref21] Islam M. M. Tani C. Watanabe-Sugimoto M. Uraji M. Jahan M. S. Masuda C.. (2009). Myrosinases, TGG_1_ and TGG_2_, redundantly function in ABA and MeJA signaling in *Arabidopsis* guard cells. Plant Cell Physiol. 50, 1171–1175. 10.1093/pcp/pcp066, PMID: 19433491

[ref22] Juntawong P. Girke T. Bazin J. Bailey-Serres J. (2014). Translational dynamics revealed by genome-wide profiling of ribosome footprints in *Arabidopsis*. Proc. Natl. Acad. Sci. U. S. A. 111, E203–E212. 10.1073/pnas.1317811111, PMID: 24367078 PMC3890782

[ref23] Kim J. Y. Kwak K. J. Jung H. J. Lee H. J. Kang H. (2010). MicroRNA402 affects seed germination of *Arabidopsis thaliana* under stress conditions via targeting DEMETER-LIKE Protein3 mRNA. Plant Cell Physiol. 51, 1079–1083. 10.1093/pcp/pcq072, PMID: 20460498

[ref24] Klepikova A. V. Kasianov A. S. Gerasimov E. S. Logacheva M. D. Penin A. A. (2016). A high resolution map of the *Arabidopsis thaliana* developmental transcriptome based on RNA-seq profiling. Plant J. 88, 1058–1070. 10.1111/tpj.13312, PMID: 27549386

[ref25] Lechner E. Leonhardt N. Eisler H. Parmentier Y. Alioua M. Jacquet H.. (2011). MATH/BTB CRL3 receptors target the homeodomain-leucine zipper ATHB6 to modulate abscisic acid signaling. Dev. Cell 21, 1116–1128. 10.1016/j.devcel.2011.10.018, PMID: 22172674

[ref26] Li S. Liua K. Zhang S. Wang X. Rogers K. Ren G.. (2017). STV1, a ribosomal protein, binds primary microRNA transcripts to promote their interaction with the processing complex in *Arabidopsis*. Proc. Natl. Acad. Sci. U. S. A. 114, 1424–1429. 10.1073/pnas.1613069114, PMID: 28115696 PMC5307444

[ref27] Liu Z. Q. Yan L. Wu Z. Mei C. Lu K. Yu Y. T. (2012). Cooperation of three WRKY-domain transcription factors WRKY18, WRKY40, and WRKY60 in repressing two ABA-responsive genes ABI4 and ABI5 in *Arabidopsis*. J. Exp. Bot. 63, 6371–6392. 10.1093/jxb/ers293, PMID: 23095997 PMC3504491

[ref28] Merchante C. Stepanova A. N. Alonso J. M. (2017). Translation regulation in plants: an interesting past, an exciting present and a promising future. Plant J. 90, 628–653. 10.1111/tpj.13520, PMID: 28244193

[ref29] Moin M. Bakshi A. Saha A. Dutta M. Madhav S. M. Kirti P. B. (2016a). Rice ribosomal protein large subunit genes and their spatio-temporal and stress regulation. Front. Plant Sci. 7:1284. 10.3389/fpls.2016.01284, PMID: 27605933 PMC4995216

[ref30] Moin M. Bakshi A. Saha A. Udaya Kumar M. Reddy A. R. Rao K. V.. (2016b). Activation tagging in indica rice identifies ribosomal proteins as potential targets for manipulation of water-use efficiency and abiotic stress tolerance in plants. Plant Cell Environ. 39, 2440–2459. 10.1111/pce.12796, PMID: 27411514

[ref31] Mustroph A. Zanetti M. E. Jang C. J. H. Holtan H. E. Repetti P. P. Galbraith D. W.. (2009). Profiling translatomes of discrete cell populations resolves altered cellular priorities during hypoxia in *Arabidopsis*. Proc. Natl. Acad. Sci. U. S. A. 106, 18843–18848. 10.1073/pnas.0906131106, PMID: 19843695 PMC2764735

[ref32] Narsai R. Law S. R. Carrie C. Xu L. Whelan J. (2011). In-depth temporal transcriptome profiling reveals a crucial developmental switch with roles for RNA processing and organelle metabolism that are essential for germination in *Arabidopsis*. Plant Physiol. 157, 1342–1362. 10.1104/pp.111.183129, PMID: 21908688 PMC3252162

[ref33] Ng L. M. Melcher K. Teh B. T. Xu H. E. (2014). Abscisic acid perception and signaling: structural mechanisms and applications. Acta Pharmacol. Sin. 35, 567–584. 10.1038/aps.2014.5, PMID: 24786231 PMC4813750

[ref34] Petryszak R. Keays M. Tang Y. A. Fonseca N. A. Barrera E. Burdett T.. (2016). Expression Atlas update—an integrated database of gene and protein expression in humans, animals and plants. Nucleic Acids Res. 44, D746–D752. 10.1093/nar/gkv1045, PMID: 26481351 PMC4702781

[ref35] Rosado A. Li R. Van de ven W. Hsu E. Raikhel N. V. (2012). *Arabidopsis* ribosomal proteins control developmental programs through translational regulation of auxin response factors. Proc. Natl. Acad. Sci. U. S. A. 109, 19537–19544. 10.1073/pnas.1214774109, PMID: 23144218 PMC3511758

[ref36] Rozen S. Skaletsky H. (2000). Primer3 on the WWW for general users and for biologist programmers. Methods Mol. Biol. 132, 365–386. 10547847 10.1385/1-59259-192-2:365

[ref37] Sah S. K. Reddy K. R. Li J. (2016). Abscisic acid and abiotic stress tolerance in crop plants. Front. Plant Sci. 7:571. 10.3389/fpls.2016.00571, PMID: 27200044 PMC4855980

[ref38] Santos Teixeira J. A. ten Tusscher K. H. (2019). The systems biology of lateral root formation: connecting the dots. Mol. Plant 12, 784–803. 10.1016/j.molp.2019.03.015, PMID: 30953788

[ref39] Shi C. Wang Y. Guo Y. Chen Y. Liu N. (2017). Cooperative down-regulation of ribosomal protein L10 and NF-κB signaling pathway is responsible for the anti-proliferative effects by DMAPT in pancreatic cancer cells. Oncotarget 8, 35009–35018. 10.18632/oncotarget.16557, PMID: 28388532 PMC5471030

[ref40] Singh K. Paul A. Kumar S. Ahuja P. S. (2009). Cloning and differential expression of QM like protein homologue from tea [*Camellia sinensis* (L.) O. Kuntze]. Mol. Biol. Rep. 36, 921–927. 10.1007/s11033-008-9264-x, PMID: 18454353

[ref41] Sormani R. Masclaux-Daubresse C. Daniele-Vedele F. Chardon F. (2011). Transcriptional regulation of ribosome components are determined by stress according to cellular compartments in *Arabidopsis thaliana*. PLoS One 6:e28070. 10.1371/journal.pone.0028070, PMID: 22164228 PMC3229498

[ref42] Wang R. S. Pandey S. Li S. Gookin T. E. Zhao Z. Albert R.. (2011). Common and unique elements of the ABA-regulated transcriptome of *Arabidopsis* guard cells. BMC Genomics 12:216. 10.1186/1471-2164-12-216, PMID: 21554708 PMC3115880

[ref43] Winter D. Vinegar B. Nahal H. Ammar R. Wilson G. V. Provart N. J. (2007). An “electronic fluorescent pictograph” browser for exploring and analyzing large-scale biological data sets. PLoS One 2:e718. 10.1371/journal.pone.0000718, PMID: 17684564 PMC1934936

[ref44] Wintermans J. F. G. M. De Mots A. (1965). Spectrophotometric characteristics of chlorophylls a and b and their phenophytins in ethanol. Biochim. Biophys. Acta 109, 448–453. 10.1016/0926-6585(65)90170-6 5867546

[ref45] Yamasaki S. Matsuura H. Demura T. Kato K. (2015). Changes in polysome association of mRNA throughout growth and development in *Arabidopsis thaliana*. Plant Cell Physiol. 56, 2169–2180. 10.1093/pcp/pcv133, PMID: 26412777

[ref46] Yoshida T. Christmann A. Yamaguchi-Shinozaki K. Grill E. Fernie A. (2019). Revisiting the basal role of ABA—roles outside of stress. Trends Plant Sci. 24, 625–635. 10.1016/j.tplants.2019.04.008, PMID: 31153771

[ref47] Zhang F. Liu S. Li L. Zuo K. Zhao L. Zhang L. (2016). Genome-wide inference of protein-protein interaction networks identifies crosstalk in abscisic acid signaling. Plant Physiol. 171, 1511–1522. 10.1104/pp.16.00057, PMID: 27208273 PMC4902594

[ref48] Zorzatto C. MacHado J. P. B. Lopes K. V. G. Nascimento K. J. T. Pereira W. A. Brustolini O. J. B.. (2015). NIK1-mediated translation suppression functions as a plant antiviral immunity mechanism. Nature 520, 679–682. 10.1038/nature14171, PMID: 25707794 PMC4779052

