# Supplementary material for: Ribosomal Protein RPL10A Contributes to Early Plant Development and Abscisic Acid-Dependent Responses in Arabidopsis
Source: Front Plant Sci. 2020 Nov 5;11:582353. doi: 10.3389/fpls.2020.582353 (PMC7674962; doi:10.3389/fpls.2020.582353)
Supplement: Supplementary file 1 [file Data_Sheet_1.pdf]

SUPPLEMENTARY DATA

SUPPLEMENTARY FIGURES LEGENDS

Supplementary Figure S1. Variation of *RPL10A*, *RPL10B* and *RPL10C* transcript levels. (**A)** Microarray data of *RPL10A*, *RPL10B* and *RPL10C* transcript levels during germination. Seeds were directly removed from the silique (Harvest), desiccated for 15 days in darkness (0 h) and stratified at 4°C in the dark for 48 h. Seeds were then transferred into continuous light and further collected at 1, 6, 12, 24 and 48 h (Narsai et al., 2011). (**B)** Developmental transcriptomic profiling of *RPL10A*, *RPL10B* and *RPL10C* genes during seed germination based on RNA-seq data (Klepikova et al., 2016). (**C)** Transcript levels of *RPL10A*, *RPL10B* and *RPL10C* genes in one-day-old seedling, based on RNA-seq data (Klepikova et al., 2016). (**D)** Microarray data of *RPL10A* transcript levels in mesophyll and guard cells without (Mock) or with 100 μM ABA treatment (Arabidopsis electronic Fluorescent Pictograph (eFP) Browser, Winter et al., 2007; Yang et al., 2008).

Supplementary Figure S2. Expression analysis of *RPL10* genes in WT, *rpl10A/+* mutants and *RPL10A*-overexpressing plants. (**A)** Transcript levels of *RPL10A, RPL10B* and *RPL10C* genes in WT and *rpl10A-1/+* after 4 h of 10 μM ABA treatment relative to control condition (without ABA, Control) (ABA/C). analyzed by RT-qPCR. *ACTIN2* was used as a gene reference. Results represent the average ± SE from three biological replicates. Different letters over the bar indicate statistical differences between samples applying ANOVA test ($P < 0.05$). **(B)** Transcript levels of *RPL10A, RPL10B* and *RPL10C* genes in WT and *rpl10A-1/+* mutant seedlings grown in MS-agar medium analyzed by RT-qPCR. *ACTIN2* was used as a gene reference. Each *RPL10* gene was normalized against *RPL10A* in WT seedlings set as 1. Different letters over the bar indicate statistical differences between samples applying ANOVA test ($P < 0.05$).

Supplementary Figure S3. Response of *rpl10A/+* mutants to exogenous ABA. **(A)** Germination percentages of WT, *rpl10A-1/+* and *rpl10A-2/+* mutants on MS-agar medium (MS) or MS-agar medium supplemented with 1 μM ABA (MS + ABA) at 72 h after stratification. Different letters over the bars indicate statistical differences between genotypes and conditions applying ANOVA test ($P < 0.05$). **(B, C)** Germination percentages of WT and *rpl10A/+* mutant seeds (*rpl10A-1/+* and *rpl10A-2/+*) on MS-agar medium supplemented with 0.5 μM (B, MS + 0.5 μM ABA) (B) or 2 μM ABA (C, MS + 2 μM ABA) (C). Results are the mean of three independent experiments ± SE (n = 150 for each biological experiment). The asterisks indicate statistical differences between WT and each mutant at each time applying Student's t-test ($P < 0.05$).

Supplementary Figure S4. Effect of ABA on cotyledon greening and seedling development. (**A)** Phenotypic comparison of WT, *rpl10A-1/+* and *RPL10A*-OE seedlings grown on MS-agar medium (MS) at 4 days after stratification or on MS-agar medium supplemented with 1 μM ABA_(MS + ABA) at 8 days after stratification. Heterozygous *rpl10A-1* mutant seedlings are indicated with asterisks. **(B)** Percentage of seedling establishment (recorded when leaf #4 is emerging) of WT, *rpl10A/+* mutants (*rpl10A-1/+* and *rpl10A-2/+*) and *RPL10A*-OE lines (*RPL10A*-OE3 and *RPL10A*-OE5) grown on MS-agar medium at 12 days after stratification. Results

represent the average from three independent experiments ± SE (n = 100 for each biological experiment). For each genotype, different letters over the bars indicate statistical differences applying ANOVA test ($P < 0.05$). Heterozygous *rpl10A-1* mutant seedlings are indicated with asterisks. (**C).** Phenotypic comparison of WT, *rpl10A* mutants (*rpl10A -1/+,* and *rpl10A-2/+*) and *RPL10A*-OE seedlings. Photographs were taken 12 days after stratification.

Supplementary Figure S5. ABA Inhibition of primary root elongation and lateral root density of WT and *RPL10A*-overexpressing seedlings. (**A)** Representative images of WT and *RPL10A*-OE seedlings grown on MS-agar plates and transferred to MS-agar medium (MS) or MS-agar medium supplemented with 10 µM ABA (MS + ABA) 4 days after the transfer. Bars = 0.5 cm. (**B)** Primary root elongation in WT and *RPL10A*-OE seedlings measured each day after the transfer. Results are the mean of three independent experiments (n = 20 for each biological experiment). For each genotype and condition (with or without 10 µM ABA), different letters over the bars indicate statistical differences applying ANOVA test ($P < 0.05$). (**C)** Average root lengths after the transfer to ABA treatment relative to control conditions (ABA/C). (**D)** LR density estimated as the number of LRs per root length unit of WT and *RPL10A*-OE seedlings. The number of LRs was determined 4 days after the transfer of 5-days-old seedlings to plates supplemented or not with ABA. Results are the mean of three independent experiments ± SE (n = 20 for each biological experiment). For each genotype and condition (MS or MS + ABA), different letters over the bars indicate statistical differences applying ANOVA test ($P < 0.05$). **(E)** LR density measured 4 days after the transfer of 5-days-old seedlings to plates supplemented with ABA relative to control conditions (ABA/C).

Supplementary Figure S6. Response of *rpl10A/+* seedlings to ABA treatment at post-germination stage**.** Seven-days-old seedlings grown in MS-agar medium were transferred to MS-agar (MS) or MS-agar medium supplemented with 5 µM ABA (MS + ABA). Ten days after the transfer, petiole length **(A)**, leaf number **(B)** were measured. **(C)** Variation of the chlorophyll (Chl) content per mg fresh weight^{-1} (FW) during ABA treatment. (**D)** Representative images of WT and *rpl10A-1/+* mutant plants at 8 (upper panel) and 10 days (lower panel) after the transfer to MS-agar medium supplemented with 5 µM ABA. Asterisks indicate heterozygous *rpl10A-1* mutant seedlings. Results are the mean of three independent experiments ± SE (n = 30 for each biological experiment). For each genotype and condition (with or without 5 µM ABA), different letters over the bars indicate statistical differences applying ANOVA test at $P < 0.05$.

Supplementary Figure S7. *In silico* analysis of Arabidopsis *RPL10A* promoter. cis-elements presented in the promoter are represented with different color. A scale covering from -1000 bp to +56 bp is showed at the top of the figure. The 5'UTR is represented in white color.

Table S1. List of primers used in this work.
Response of *rpl10A/+* seedlings to ABA treatment at post-germination stage.

SUPPLEMENTARY REFERENCES

Yang Y., Costa A., Leonhardt N., Siegel R.S., Schroeder J.I., (2008). Isolation of a strong Arabidopsis guard cell promoter and its potential as a research tool. Plant Methods 19, 4-6.

Figure S1. Variation of *RPL10A*, *RPL10B* and *RPL10C* transcript levels.

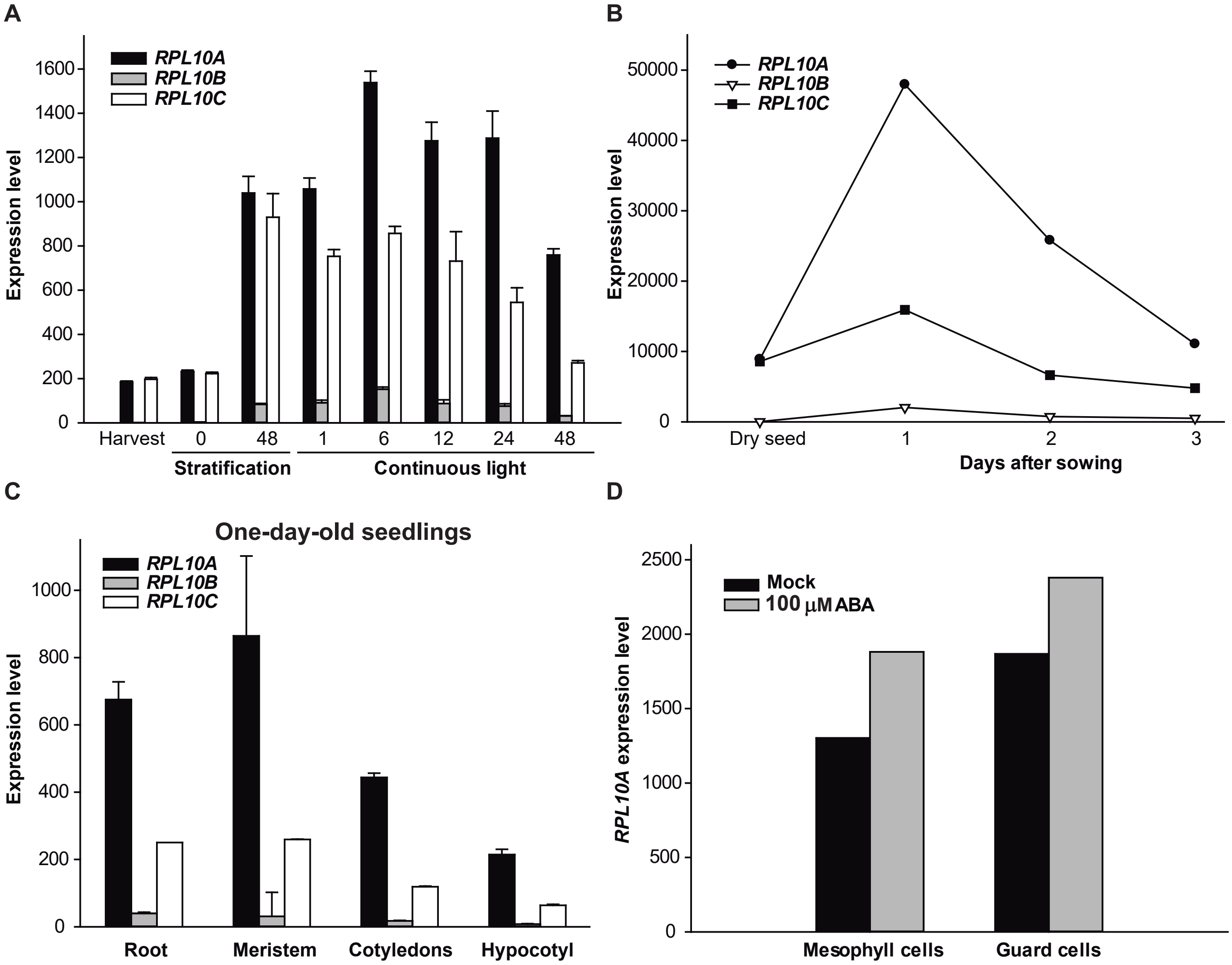

Figure S2. Expression analysis of *RPL10* genes in WT, *rpl10A-1/+* mutants and *RPL10A*-overexpressing plants.

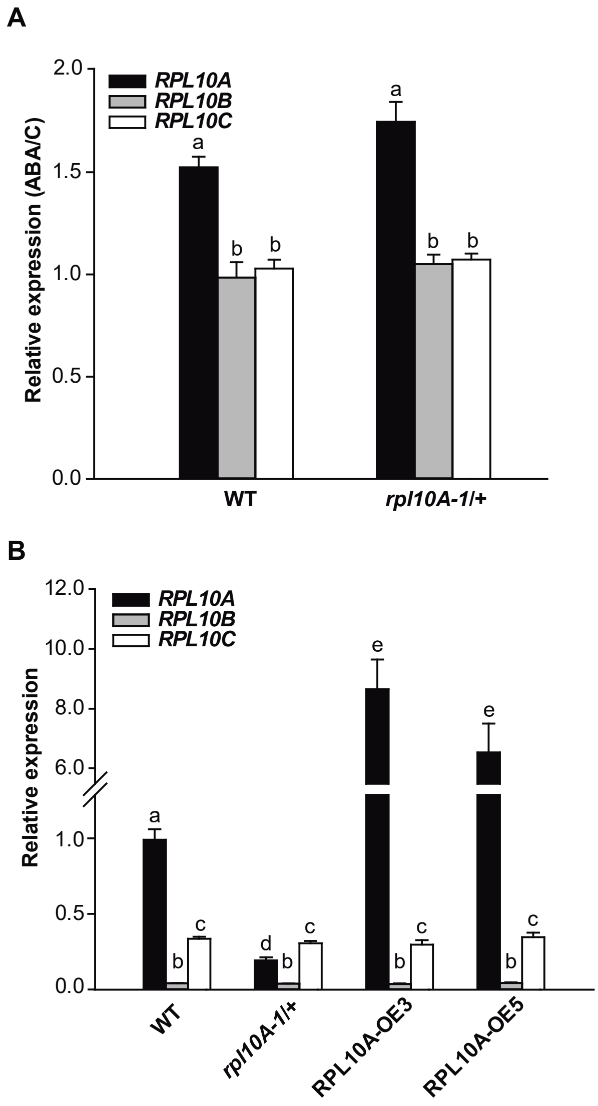

Figure S3. Response of *rpl10A/+* mutants to exogenous ABA.

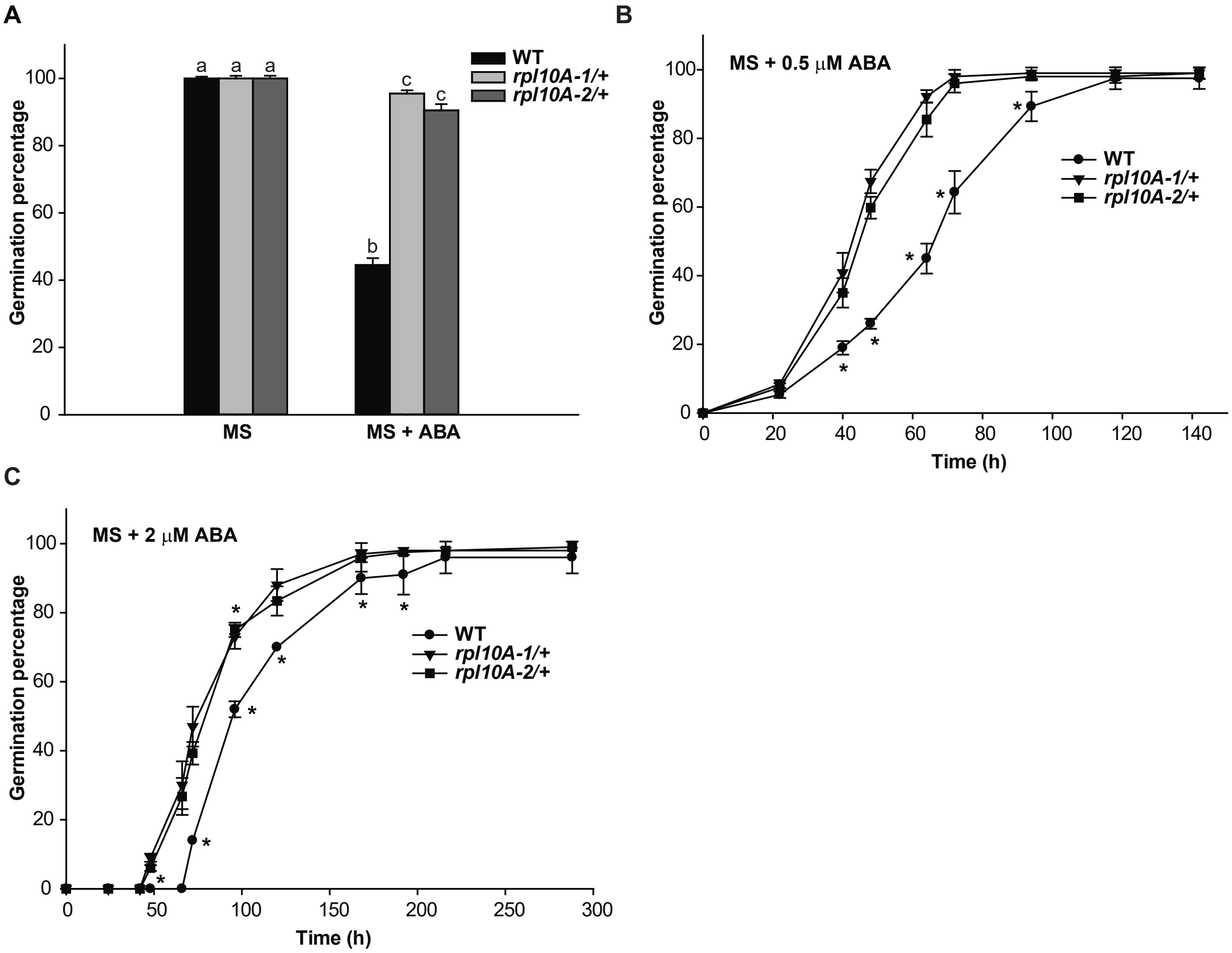

Figure S4. Effect of ABA on cotyledon greening and seedling development.

A

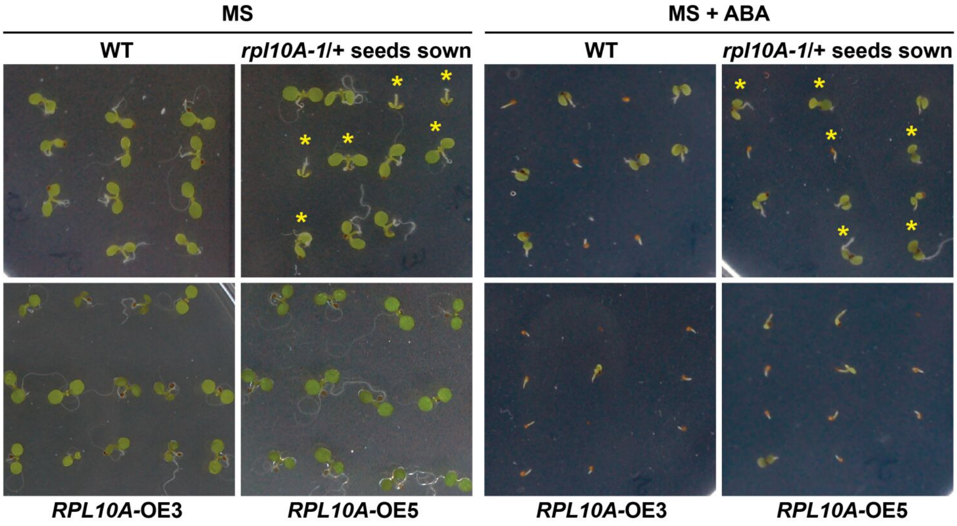

B

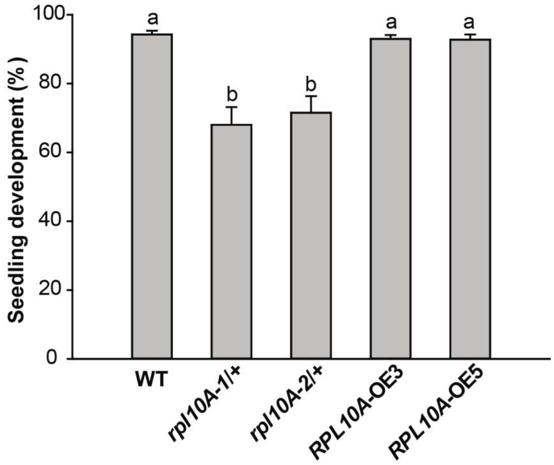

C

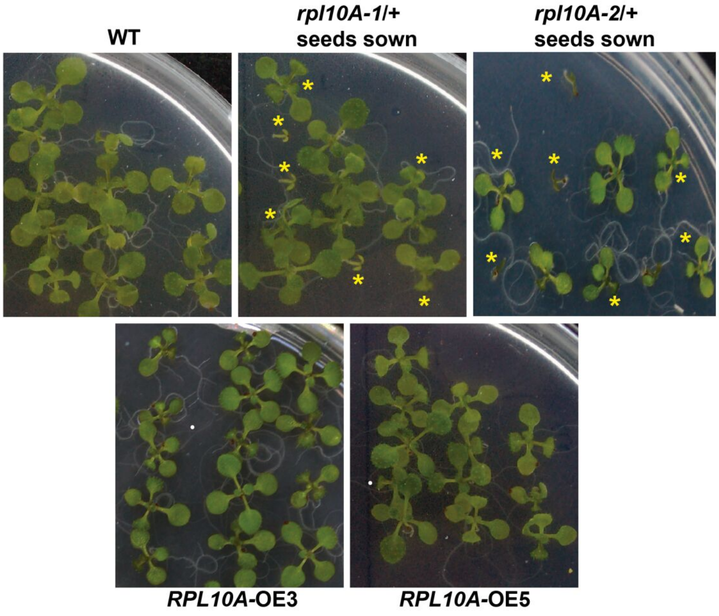

Figure S5. ABA Inhibition of primary root elongation and lateral root density of WT and *RPL10A*-overexpressing seedlings.

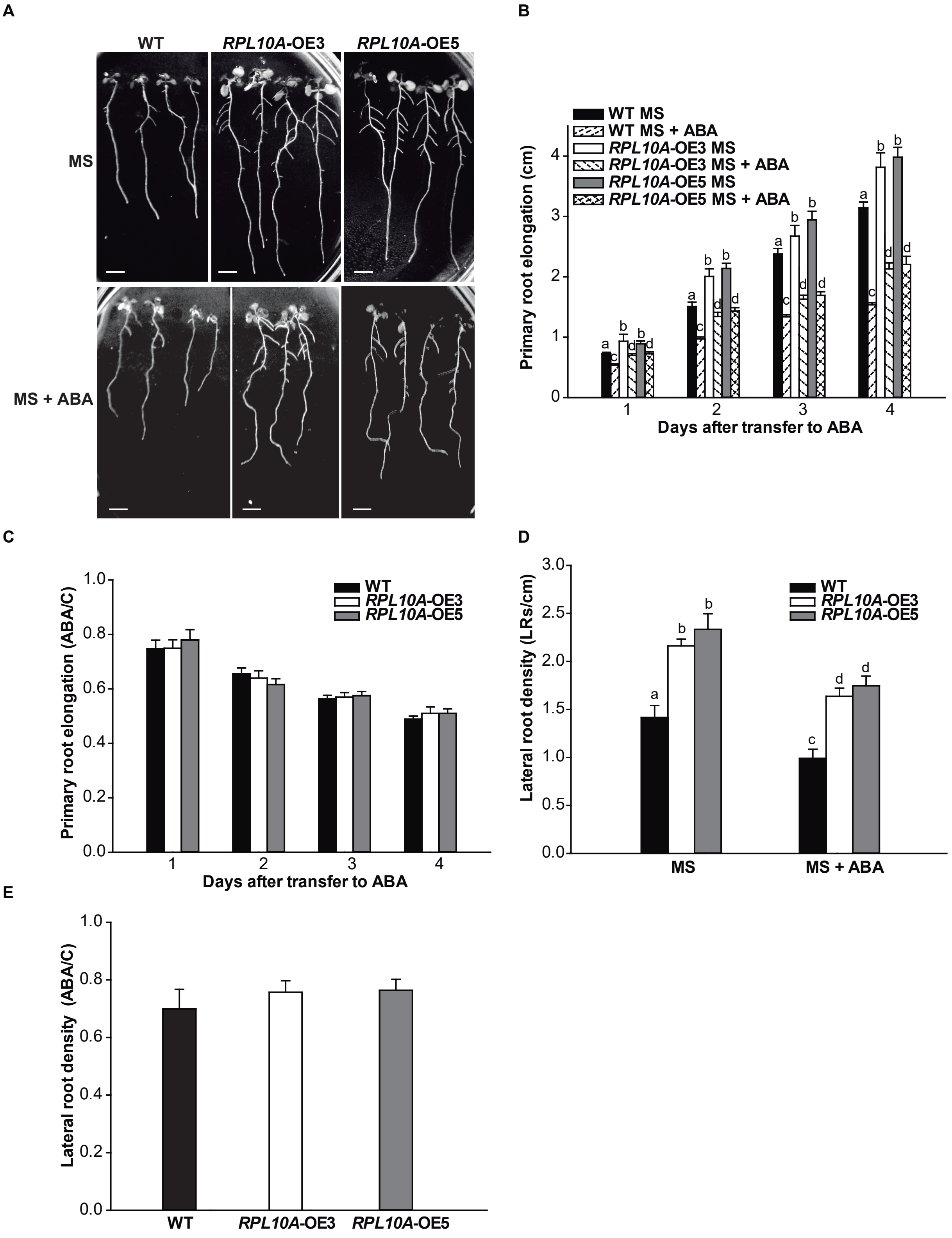

Figure S6. Response of *rpl10A*/+ seedlings to ABA treatment at post-germination stage.

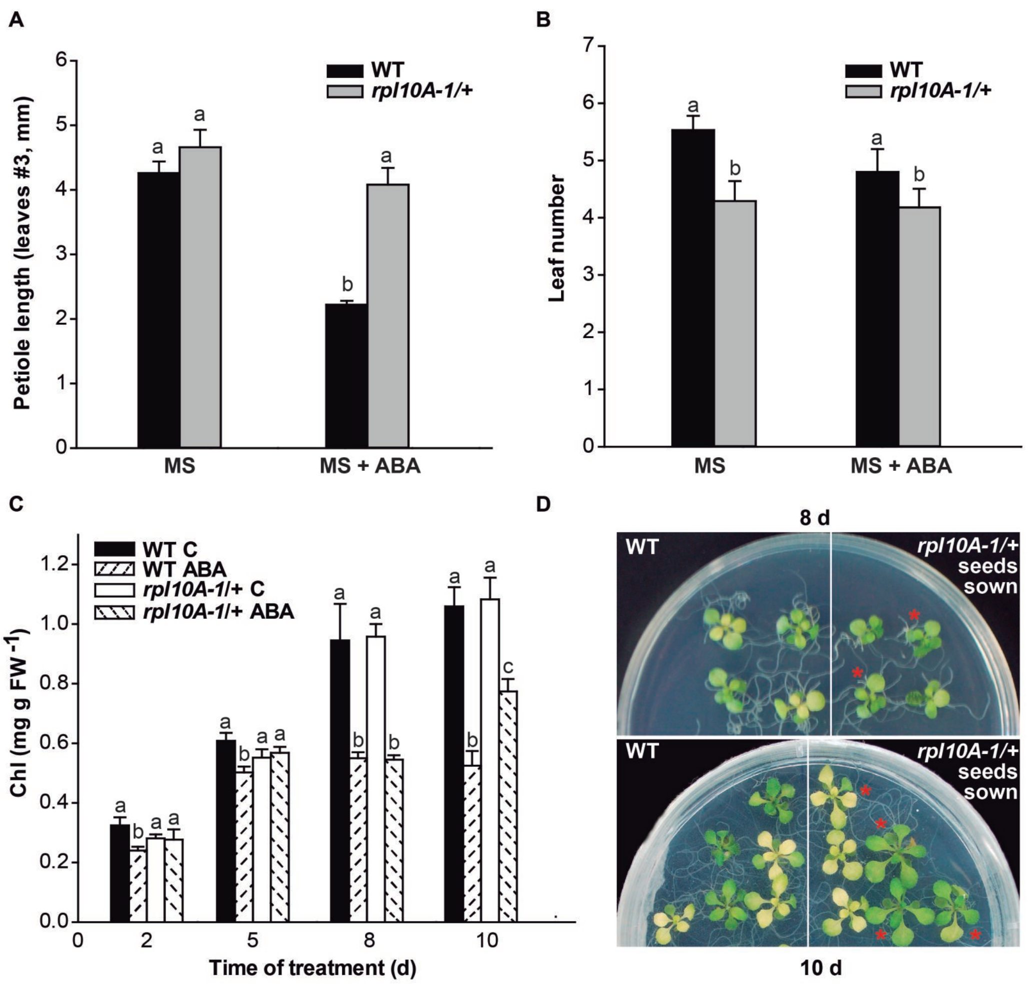

Figure S7. *In silico* analysis of Arabidopsis *RPL10A* promoter.

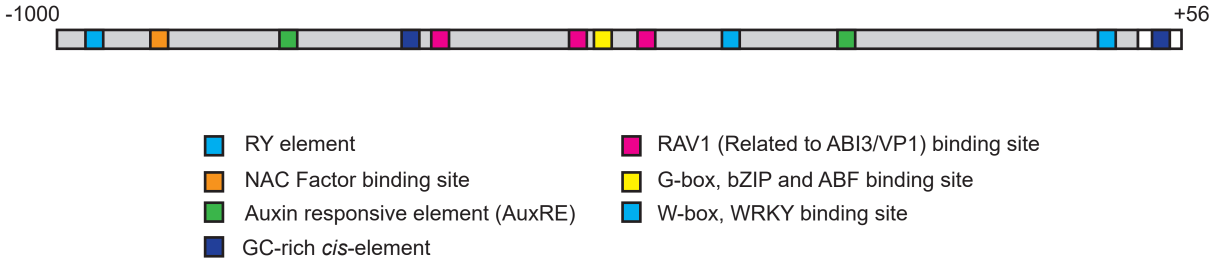

Table S1. List of primers used in this work.

Primer name	Sequence	Purpose
F-*RPL10A-1*	5'TACGATGTTGGTATGAAGAG3'	Screening of heterozygous mutants
R-*RPL10A*-1	5'TCACCGGAATGAGAAGGA3'	Screening of heterozygous mutants
L-LBSALK	5'TGGTTCACGTAGTGGGCCATCG3'	Screening of heterozygous mutants
F-*RPL10A*-RT	5'TCCTTCTCATTCCGGTGA3'	RT-qPCR
R-*RPL10A*-RT	5'GCCAAGAACGAAGGAACA3'	RT-qPCR
F-*RPL10A*-RT2	5'CAGGAAATGGGGCTTCACGAAG3'	RT-qPCR (OE lines)
R-*RPL10A*-RT2	5'GTAGTGGGCTGGCAAAAAGGC3'	RT-qPCR (OE lines)
F-*RPL10B*-RT	5'TCCCTGGTCGTCAAAAGA3'	RT-qPCR
R-*RPL10B*-RT	5’AGCACCAGCTGACAAGAA3'	RT-qPCR
F-*RPL10C*-RT	5’CCAACACCGAAGATCCAA3'	RT-qPCR
R-*RPL10C*-RT	5'TTTTGGGATCTGGCACAC3'	RT-qPCR
F-35S_{prom}	5'GAGGAGCATCGTGGAAAAAGA3'	Screening of OE lines
L-*ACTIN2*	5'CAGATGCCCAGAAGTCTTGTTC3'	RT-qPCR
R-*ACTIN2*	5'AACGACCTTAATCTTCATGCTGC3'	RT-qPCR
F-*Kpn*I-R*PL10A*	5’GACTAGGTACCATGGAAGAAGACCTGCG3’	Cloning
R-*Sal*I-*RPL10A*	5’TGTCAGTCGACTCAGTAGTGGGCTGGCAA3’	Cloning
